# PARP Inhibitors and Myeloid Neoplasms: A Double-Edged Sword

**DOI:** 10.3390/cancers13246385

**Published:** 2021-12-20

**Authors:** Clifford M. Csizmar, Antoine N. Saliba, Elizabeth M. Swisher, Scott H. Kaufmann

**Affiliations:** 1 Department of Medicine, Mayo Clinic, Rochester, MN 55905, USA; csizmar.clifford@mayo.edu; 2 Division of Hematology, Department of Medicine, Mayo Clinic, Rochester, MN 55905, USA; saliba.antoine@mayo.edu; 3 Division of Medical Oncology, Department of Oncology, Mayo Clinic, Rochester, MN 55905, USA; 4 Division of Gynecologic Oncology, Department of Obstetrics and Gynecology, University of Washington, Seattle, WA 98195, USA; swishere@uw.edu; 5 Department of Molecular Pharmacology and Experimental Therapeutics, Mayo Clinic, Rochester, MN 55905, USA; 6 Division of Oncology Research, Department of Oncology, Mayo Clinic, Rochester, MN 55905, USA

**Keywords:** PARP inhibitors, acute myeloid leukemia, myelodysplastic syndrome, myeloid neoplasms, secondary malignancies, DNA damage repair, base excision repair, non-homologous end-joining, synthetic lethality

## Abstract

**Simple Summary:**

Poly(ADP-ribose) polymerase (PARP) inhibitors, which are medications approved to treat various solid tumors, including breast, prostate, ovarian, and prostate cancers, are being examined in hematological malignancies. This review summarizes the potential role of PARP inhibitors in the treatment of myeloid diseases, particularly acute myeloid leukemia (AML). We review ongoing clinical studies investigating the safety and efficacy of PARP inhibitors in the treatment of AML, focusing on specific molecular and genetic AML subgroups that could be particularly sensitive to PARP inhibitor treatment. We also discuss reports describing an increased risk of treatment-related myeloid neoplasms in patients receiving PARP inhibitors for solid tumors.

**Abstract:**

Despite recent discoveries and therapeutic advances in aggressive myeloid neoplasms, there remains a pressing need for improved therapies. For instance, in acute myeloid leukemia (AML), while most patients achieve a complete remission with conventional chemotherapy or the combination of a hypomethylating agent and venetoclax, de novo or acquired drug resistance often presents an insurmountable challenge, especially in older patients. Poly(ADP-ribose) polymerase (PARP) enzymes, PARP1 and PARP2, are involved in detecting DNA damage and repairing it through multiple pathways, including base excision repair, single-strand break repair, and double-strand break repair. In the context of AML, PARP inhibitors (PARPi) could potentially exploit the frequently dysfunctional DNA repair pathways that, similar to deficiencies in homologous recombination in *BRCA*-mutant disease, set the stage for cell killing. PARPi appear to be especially effective in AML with certain gene rearrangements and molecular characteristics (*RUNX1-RUNX1T1* and *PML-RARA* fusions, *FLT3-* and *IDH1*-mutated). In addition, PARPi can enhance the efficacy of other agents, particularly alkylating agents, TOP1 poisons, and hypomethylating agents, that induce lesions ordinarily repaired via PARP1-dependent mechanisms. Conversely, emerging reports suggest that long-term treatment with PARPi for solid tumors is associated with an increased incidence of myelodysplastic syndrome (MDS) and AML. Here, we (i) review the pre-clinical and clinical data on the role of PARPi, specifically olaparib, talazoparib, and veliparib, in aggressive myeloid neoplasms and (ii) discuss the reported risk of MDS/AML with PARPi, especially as the indications for PARPi use expand to include patients with potentially curable cancer.

## 1. Introduction

Over the past decade, poly(ADP-ribose) polymerase (PARP) inhibitors (PARPi) have been investigated extensively in solid tumors and approved for use in subsets of patients with ovarian, breast, prostate, and pancreatic cancer [1,2,3,4,5,6]. These agents are especially active in cells with impaired ability to repair DNA double-strand breaks (DSBs) through the homologous recombination (HR) pathway, a high-fidelity repair pathway that is operative in the S and G2 phases of the cell cycle [7,8]. Cells with HR deficiency (HRD), e.g., cells with mutations in the tumor suppressors *BRCA1* or *BRCA2*, have diminished HR-mediated repair [9] and are more dependent on alternative, lower fidelity repair pathways such as non-homologous end-joining (NHEJ) and alternative end-joining (alt-EJ) to prevent the lethal effects of DSBs [10,11]. This lack of high fidelity DSB repair and reliance on alternative, more error-prone pathways in HRD neoplasms sets the stage for the lethal effects of PARP inhibitors (PARPi) in certain subtypes of breast, ovarian, and prostate cancer [7,8,12], especially tumors with mutations or silencing of *BRCA1/2*, *RAD51*, *RAD54*, *DSS1*, *RPA1*, *NBS1*, *ATR*, *ATM*, *CHK1*, *CHK2*, *FANCD2*, *FANCA*, or *FANCC* [12,13,14,15]. While the role of PARPi has been explored in myeloid neoplasms, PARPi have not shown consistent benefit and thus are not currently approved by the United States Food and Drugs Administration (FDA) for these disorders. At the same time, reports have emerged detailing a higher incidence of myeloid neoplasms in patients with solid tumors treated with PARPi [16,17,18,19]. Thus, PARPi appear to be a double-edged sword when it comes to myeloid neoplasms.

The PARP superfamily consists of 18 proteins encoded by different genes but sharing a conserved C-terminal catalytic domain that transfers adenosine diphosphate (ADP)-ribose moieties to various acceptors. Among those 18 PARP superfamily proteins, PARP1, PARP2, and PARP3 can be stimulated by DNA strand breaks [20]. After binding to nicked DNA through its N-terminal zinc fingers, PARP1, the most abundant of the superfamily members, acts on the substrate nicotinamide adenine dinucleotide (NAD^+^) to transfer ADP-ribose from nicotinamide to protein substrates [21], thereby leading to mono- or poly(ADP-ribosyl)ation (PARylation) of multiple protein substrates involved in RNA processing, DNA replication, transcription, and the DNA damage response (DDR) [22,23,24]. Much of the polymer is covalently bound to PARP1 itself. The poly(ADP-ribose) (pADPr) polymers recruit hundreds of nuclear proteins, including additional DNA repair proteins such as meiotic recombination 11 (MRE11) and Nijmegen breakage syndrome (NBS1), to the SSBs [25]. The pADPr chains, which are highly negatively charged, also diminish the affinity of PARP1 for DNA, resulting in dissociation of PARP1 that is mandatory for completion of DNA repair [26,27]. In that capacity, PARPi have been shown to impair repair via inhibition of pADPr formation and through trapping of lethal PARP-DNA complexes [28].

Olaparib, the first FDA-approved PARPi, serves as a prototypical example of the various clinical applications of this drug class. This agent is approved for patients with germline *BRCA*-mutated, HER2-negative breast cancer in the metastatic setting [13]; for advanced ovarian cancer as first-line maintenance therapy, with bevacizumab for HRD tumors, and without bevacizumab in the context of germline or somatic *BRCA* mutations [29,30]; as maintenance therapy for recurrent ovarian cancer after partial or complete response to platinum-based therapy regardless of HR or *BRCA* status [18,31]; and as maintenance therapy for metastatic pancreatic cancer with germline *BRCA* mutations [32]. In addition, olaparib is associated with prolonged imaging-based progression-free survival and overall survival in men with metastatic castration-resistant prostate cancer with at least one alteration in *BRCA1*, *BRCA2*, or *ATM* [33,34].

Conversely, *BRCA1/2* and *ATM* mutations are not common in myeloid malignancies [35]. However, many myeloid neoplasms possess dysregulated HR mechanisms, defective DDR pathways, or chromosomal instability [36,37,38,39,40,41,42], suggesting the possibility that PARPi might be active in these malignancies. The fact that chromosomal aberrations are typically associated with chemotherapy resistance has suggested a subgroup of patients with acute leukemia in whom PARP inhibition might be most promising [43,44]. Accordingly, there has been substantial interest in exploring the therapeutic potential of PARP inhibition in various myeloid neoplasms, including acute myeloid leukemia (AML), myelodysplastic syndrome (MDS), myeloproliferative neoplasms (MPN), and others. In this review, we highlight (i) the preclinical data supporting the use of PARPi in myeloid neoplasms, (ii) the current clinical experience using PARPi in myeloid diseases, and (iii) the recent recognition of therapy-emergent myeloid neoplasms in patients with solid tumors who were treated with PARPi.

## 2. Chemical Biology of PARP Inhibitors

### 2.1. Structure and Function of ADP-Ribosyltransferases

PARP1 is the founding member of the PARP family of proteins [20,45]. PARP1 is an (ADP-ribosyl)transferase that catalyzes the transfer of multiple ADP-ribose units to polypeptides using NAD^+^ as a substrate to generate pADPr polymers. This PARylation of target proteins has been associated with a multitude of cellular processes, including the maintenance of genomic integrity [46,47], regulation of gene transcription [48,49,50], protein stabilization or degradation [51,52,53], and modulation of cellular metabolism [54,55]. However, the most widely recognized function of PARP1 is its role in the cellular response to DNA damage as a mediator of base excision repair (BER) and HR [56,57,58,59,60].

**Figure 1:**
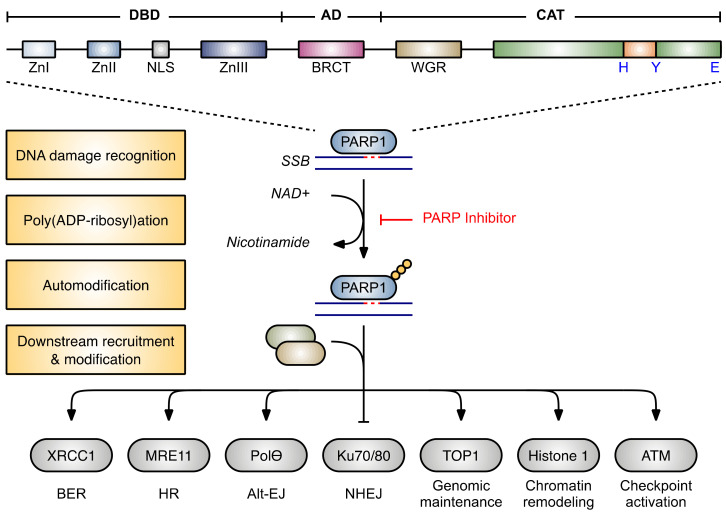
Structure and Function of PARP1. PARP1 comprises a DNA-binding domain (DBD), automodification domain (AD), and catalytic domain (CAT). The DBD contains three zinc finger motifs (Zn) that recognize sites of DNA damage. A nuclear localization signal (NLS) retains PARP1 within the nucleus. The AD contains a BRCA1 carboxy-terminal (BRCT) domain that enables the recruitment and scaffolding of downstream proteins. The CAT houses a Trp-Gly-Arg (WGR) motif that stabilizes DNA binding as well as the His-Tyr-Glu (HYE) catalytic triad. Once bound to DNA, a conformational change activates the CAT to catalyze the poly(ADP)-ribosylation (PARylation) of PARP1 within the AD. PARylation proceeds by transferring ADP-ribose moieties from nicotinamide adenine dinucleotide (NAD^+^) to the acceptor polypeptide. The resulting pADPr chains recruit other DNA repair proteins; and PARylation of addition protein substrates helps elicit a variety of actions related to DNA repair, genomic maintenance, transcription, and cell cycle progression. Abbreviations: TOP1, DNA topoisomerase I; BER, base excision repair; HR, homologous recombination; Alt-EJ, alternative end-joining; NHEJ, non-homologous end-joining. Figure adapted from Rouleau, M. et al. [82].

PARP1 is a nuclear protein comprised of three functional domains (Figure 1): (1) an *N*-terminal DNA-binding domain (DBD); (2) a central automodification domain (AD); and (3) a *C*-terminal catalytic domain (CAT) [61,62]. The DBD contains three zinc finger motifs (Zn) that facilitate the sequence-independent recognition of DNA single strand breaks (SSBs) and DSBs [63,64]. The Zn1 and Zn2 domains are structural homologues that are similar to the DBD of DNA polymerase III [65]. Despite their structural similarity, however, Zn1 and Zn2 exhibit divergent biochemical activities. The Zn1 domain is responsible for driving the activating conformational change in the CAT, as deletion of the Zn1 domain ablates PARylation activity but preserves DNA binding affinity [66,67]. In contrast, the Zn2 domain is primarily responsible for high-affinity DNA interactions, as Zn2 deletion reduces DNA-binding affinity but has minimal impact on the detection of pADPr polymers [67,68,69]. Meanwhile, the structurally distinct Zn3 domain collaborates with Zn1 and the conserved Trp-Gly-Arg (WGR) motif in the CAT to bind to DSBs, interfacing with both the 3′- and 5′-terminated DNA strands via sequence-independent interactions with the ribose-phosphate backbone of the DNA [61,62].

The AD contains Lys and Glu residues that serve as ADP-ribose acceptors [66,70], enabling the PARylation of PARP1 via both internal self-modification of monomeric PARP1 and trans-PARylation of dimeric PARP1 [62,67,71,72]. Modification of a third set of amino acid sidechains (serine residues) [73] has been more recently described and appears to occur predominantly when PARP1 is complexed with its binding partner HPF1 [74,75]. The AD also contains a BRCA1 carboxy-terminal motif (BRCT) that facilitates the recruitment of PAR-binding proteins and assembly of DNA repair machinery [76,77,78].

Finally, the CAT contains a distinct His-Tyr-Glu (HYE) motif, where the His and Tyr residues position the NAD^+^ substrate in the orientation required for the Glu to catalyze the ADP-ribose transfer to the accepting polypeptide [79,80]. Indeed, this HYE motif is conserved across the PARP family members capable of performing true poly(ADP-ribose) transfer, while loss of the Glu residue renders the enzyme capable of catalyzing only a single mono(ADP-ribose) transfer event [81]. The essential WGR motif is also housed within the CAT, where it binds to the 5′-terminus of the DNA break to extend the contacts made by the Zn1 ‘base stacking loop’ [61].

PARP1 has been most extensively studied in the context of DNA repair. PARP1 is rapidly recruited to sites of SSBs and DSBs, where, upon binding to the damaged DNA, its catalytic activity is increased up to 500-fold [62]. This drives the synthesis of long, branched strands of PARP1-bound pADPr, which recruits other DNA repair proteins [61,83] such as the scaffolding protein X-ray cross complementing protein 1 (XRCC1), which mediates BER, as well as MRE11, which drives HR [25,57,58]. PARP1 also PARylates BRCA1 to further regulate HR [78], competes with Ku proteins to suppress error-prone NHEJ [84], and is essential for microhomology mediated repair via the alt-EJ pathway [85,86]. Finally, PARP1 plays a critical role in stabilizing replication forks that have stalled after encountering obstructing DNA lesions [77,87]. Thus, PARP1 plays a multifaceted role in responding to DNA damage.

Seventeen other PARP family members have been identified based on their structural homology to PARP1 [45]. Of these, however, only six (PARPs 1-4 and tankyrases 1-2) are thought to catalyze the formation of pADPr polymers, whereas the remainder perform only mono(ADP-ribosyl)ation [45,82]. Thus, there are efforts to refer to the PARP family more generally as ADP-ribosyl transferase diphtheria toxin-like (ARTD) proteins [88], although this nomenclature has not yet been universally adopted. As with PARP1, PARP2 and PARP3 are DNA-dependent PARPs with roles in DNA repair. PARP2 binds damaged DNA, catalyzes PARylation, and displays automodification properties similar to PARP1 [89]. It also localizes to the nucleus, where it may account for the residual PARylation activity seen in PARP1 deficient cells. PARP2 also collaborates with PARP1 to recruit XRCC1, DNA polymerase β, and DNA ligase III to mediate BER [72]. PARP3 acts in concert with PARP1/2 to respond to DSBs, stabilizes the mitotic spindle, and helps maintain telomere integrity [90,91]. Importantly, due to the structural similarity between these family members, PARPi exhibit substantial activity against each, and this promiscuity may account for the varied biologic effects of these agents [92,93].

### 2.2. Proposed Mechanisms of Synthetic Lethality in HRD

Early work demonstrated that HR-deficient cells were exquisitely sensitive to PARP inhibition [7,8]. In the absence of PARP1, replication forks stall and collapse upon encountering DNA SSBs, necessitating repair by HR [8]. In *BRCA1/2*-mutant cells with defective HR machinery and inhibited PARP1, the collapsed replication forks become largely irreparable, resulting in chromosomal instability, cell cycle arrest, and subsequent apoptosis [7]. Similar results have since been observed with mutations in other DNA repair proteins, including ataxia telangiectasia mutated (ATM) [94] and members of the Fanconi anemia pathway [15]. Moreover, PARP inhibition sensitizes HR-competent cells to certain types of DNA damage, highlighting the variegated roles of PARP in maintaining genomic integrity [95,96]. Several models have been proposed to describe the observed synthetic lethality between HR deficiency and PARP inhibition [97]: inhibition of BER, PARP trapping on DNA, impaired recruitment of BRCA1, activation of NHEJ, inhibition of alt-EJ, and destabilization of stalled replication forks. However, each model has its limitations, and no single model fully explains the spectrum of PARPi activity. As such, the true mechanism is likely multifactorial [10].

#### 2.2.1. Inhibition of Base Excision Repair

**Figure 2:**
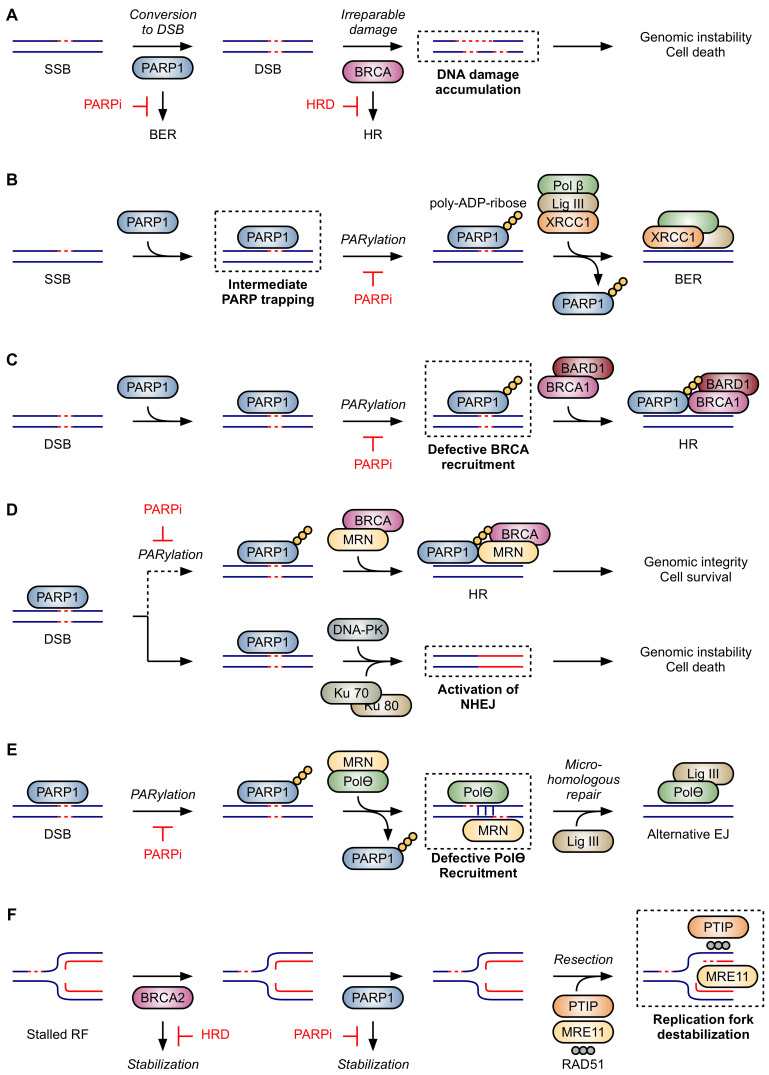
Proposed Mechanisms of Synthetic Lethality with PARP Inhibitors. (**A**) Inhibition of base excision repair (BER). Under physiologic conditions, DNA single-strand breaks (SSBs) are repaired via BER in a process that depends on PARP enzymes. When PARP is inhibited, SSBs can be converted to double-strand breaks (DSBs), which can be repaired through homologous recombination (HR). In cells with deficient HR mechanisms—such as inactivating mutations in *BRCA1/2* or *RAD51*—concurrent PARP inhibition renders the cells incapable of performing high-fidelity repair. Thus, DNA damage accumulates, ultimately leading to cell death. (**B**) PARP trapping. PARP catalytic activity is required for the auto-modification of PARP1 with covalently bound pADPr groups (PARylation). This automodification both recruits other proteins and decreases the affinity of PARP for the damaged DNA. PARP inhibitors (PARPi) impair PARylation, rendering PARP1 and PARP2 unable to efficiently dissociate from damaged DNA. This “traps” PARP on the DNA and impairs the recruitment and assembly of downstream repair machinery. (**C**) Impaired BRCA1 recruitment. PARP is also present at sites of DSBs, where PARP automodification recruits the BARD1/BRCA1 complex. By inhibiting PARylation, PARPi prevent effective recruitment of BRCA1 and thus impede HR. (**D**) Activation of non-homologous end-joining (NHEJ). PARP automodification favors HR by recruiting members of the MRN (MRE11, RAD51, NBS1) complex and BRCA1/2 proteins, which compete with the proteins Ku70 and Ku80 that facilitate error-prone NHEJ. PARP inhibition derepresses NHEJ by preventing the rapid recruitment of HR proteins and allowing recruitment of Ku70 and Ku80, thereby permitting error-prone NHEJ and expediting the accumulation of lethal genomic alterations. (**E**) Defective Polθ recruitment. PARP1 activity recruits the MRN complex and Polθ to promote microhomology-mediated repair via alternative end-joining. HR-deficient tumors are heavily reliant on Polθ activity, and PARP inhibition impairs effective recruitment to DSBs. (**F**) Destabilization of stalled replication forks (RFs). BRCA2 helps stabilize and rescue stalled RFs by enabling homology-driven repair to bypass the obstructing lesion. Loss of BRCA2 leads to reliance on PARP activity for stabilization of stalled RFs. PARPi prevent this stabilization to promote PTIP (PAX transcription activation domain interacting protein) and MRE11-mediated RF resection and genomic instability. The final common pathway of all mechanisms is the accumulation of unrepaired DNA damage, resultant loss of genomic integrity, and ultimately, cell death.

PARPi were first thought to exert their cytotoxic efficacy in HR-deficient cells through the inhibition of BER (Figure 2A). BER is the primary method by which cells repair DNA SSBs, and PARP1 is essential for this process [56,98]. In the absence of PARP1, SSBs were thought to be converted to DSBs and repaired by HR. HR-deficient cells are unable to complete this “backup” repair step, forcing the DSBs to either be repaired by low-fidelity processes such as NHEJ or be left unrepaired, leading to genomic instability and cell death [99]. This mechanism was thought to be responsible for the synthetic lethality observed when PARP is inhibited in HR-deficient cells and malignancies [7,8,100]. Critical experiments, however, failed to support this model. While PARP1 is hyperactivated in BRCA1/2-deficient cells, treating these cells with PARPi did not yield a detectable accumulation of SSBs as was expected [101]. Moreover, while PARPi were clearly cytotoxic to HR-deficient cells, knockdown of the protein immediately downstream of PARP1 in the BER pathway—XRCC1—was not lethal, further suggesting an alternative mechanism [10]. Finally, neither the knock-down of PARP1 itself in HR-competent cells [28] nor its knock-out in mice [57,102] was lethal. Collectively, these observations prompted investigators to look further to find an explanation for the synthetic lethality of HR deficiency and PARPi treatment.

#### 2.2.2. PARP Trapping

The observed synergy between PARPi and DNA-damaging agents suggests that PARP1 becomes “trapped” at DNA lesions (Figure 2B) [28,103,104]. The Zn1 and Zn2 domains of native PARP1 have a high affinity for exposed nucleotide bases and contiguous regions of phosphate backbone, common features at sites of DNA damage [61,67]. Upon binding to DNA, PARP1 undergoes a conformation change that increases the activity of the catalytic domain up to 500-fold, facilitating the auto-PARylation of DNA-bound PARP1 [62,82]. This automodification simultaneously recruits downstream DNA repair proteins and decreases PARP1’s affinity for the damaged DNA, allowing the recruited repair proteins to bind to the DNA lesion and displace PARP1 [27,58,72,98,105]. Because PARPi inhibit auto-PARylation, they preserve the high affinity PARP1–DNA interaction and leave PARP1 trapped at the DNA lesion, where it sterically inhibits the binding of subsequent repair proteins. This notion is supported by experiments demonstrating that overexpression of the isolated DNA binding domain of PARP1 (in the absence of the catalytic domain) potentiates alkylation-induced DNA damage [106,107]. Moreover, PARP trapping was demonstrated to be more cytotoxic than inhibition of BER through PARP knockdown, indicating that the presence of PARP1 is required to realize the full cytotoxicity of PARP inhibition in HR-competent cells [28]. In contrast, PARP1 knockdown is sufficient to induce lethality in BRCA1/2-deficient cells—an observation that casts doubt on PARP trapping as the sole explanation for cytotoxicity of PARPi in HR-deficient cells [7,10].

#### 2.2.3. Impaired Recruitment of BRCA1

In response to DSBs, a serine residue of the histone protein H2AX becomes rapidly phosphorylated to form γH2AX [108]. While it was originally thought that BRCA1 was recruited to DSBs by γH2AX, subsequent studies revealed that γH2AX serves primarily to stabilize BRCA1 at DSBs rather than recruit it [109]. Instead, BRCA1 forms a heterodimer with the PAR-binding protein BARD1, which facilitates BRCA1 recruitment at sites of PARylation [76]. Thus, when *BRCA1*-mutant cells are exposed to PARPi, both BRCA1 recruitment and stabilization at the site of DNA damage are impaired (Figure 2C). While this model provides a rationale for the exquisite efficacy of PARPi in cells with certain *BRCA1* mutations, it is not likely to account for the efficacy of PARPi in cells with mutational deficits in other components of the HR machinery [76].

#### 2.2.4. Activation of NHEJ

NHEJ is an error-prone method of repairing DSBs when HR cannot be utilized and is associated with high rates of mutations and chromosomal translocations [110]. PARP1 has been shown to localize to sites of DSBs, where it recruits the MRN complex—composed of MRE11, RAD50, and NBS1—and BRCA proteins [11,87]. This recruitment competes with the assembly of proteins that would otherwise drive NHEJ, such as Ku70, Ku80, and the DNA-dependent protein kinase (DNA-PK) complex (Figure 2D) [84,111]. Accordingly, PARP1/2 null cells exhibit reduced levels of HR and enhanced levels of NHEJ [112]. Similarly, deficiencies in other DSB repair pathways, such as the alternative end-joining pathway that relies up on polymerase θ, also confer sensitivity to PARPi, presumably by forcing repair through NHEJ [113]. Moreover, inactivation of NHEJ confers resistance to PARPi in HR-deficient cells [10,114]. However, this model does not account for the source of DNA damage that would be needed to produce the DSBs that drive PARPi sensitivity.

#### 2.2.5. Inhibition of Alt-EJ

Alt-EJ—also known as microhomology-mediated end-joining (MMEJ)—uses small regions of 15–100 nucleotide overhangs to repair DSBs [110]. Alt-EJ is an incompletely understood mechanism of DNA repair that requires DNA polymerase θ (Polθ), the MRN complex, and likely PARP1 [85]. As in HR, PARP1 and the MRN complex compete with Ku proteins for broken DNA ends. When possible, the MRN complex processes the exposed DNA ends to generate short regions of microhomologous overhangs [110]. PARP1 activity also recruits Polθ [115], which stabilizes the annealing of microhomologous regions, fills the resected gaps, and enables ligation by DNA ligase I or III [116]. HR deficient cells are reliant on Polθ and the alt-EJ pathway for survival, and loss of both the HR and alt-EJ pathways in vivo resulted in embryonic lethality in *Fancd2^−/−^Polq^−/−^* mice [113]. Moreover, HR-deficient cells are sensitive to Polθ depletion or inhibition both in vitro and in vivo, and exquisite synergy is seen with simultaneous PARP inhibition [113,117]. Indeed, in a murine xenograft model of HR-deficient tumors with inducible Polθ deficiency, treatment with PARPi both significantly reduced tumor volume and extended survival compared to Polθ depletion alone [113]. Similar results were obtained using the small-molecule inhibitor of Polθ, novobiocin, and the combination of novobiocin and olaparib continued to exhibit synergistic efficacy even in HR deficient, PARPi-resistant murine xenografts [117]. Collectively, these data suggest that HR deficient tumors have increased dependence on repair processes involving Polθ, which is recruited to DSBs by PARP1 (Figure 2E).

#### 2.2.6. Destabilization of Stalled Replication Forks

In addition to their roles in DNA repair, both PARP1 and BRCA2 play critical roles in restarting stalled replication forks (RFs) during periods of replication stress [118]. BRCA2 localizes to stalled replication forks, where it protects the nascent strand of DNA from degradation by stabilizing filaments of RAD51 [119]. Separately, PARP1 also recognizes stalled RFs, where it is presumed to generate pADPr polymers that recruit other repair proteins and physically impair the binding of degradative exonucleases, such as MRE11 [120,121,122]. In the absence of BRCA2 or PARP1, however, nascent DNA strands are degraded by MRE11 until the obstructing lesion has been resected, and the residual DNA is subject to non-homologous repair [120]. Thus, according to this model shown in Figure 2F, BRCA2-deficient cells are reliant upon PARP1 to stabilize stalled RFs [120], and treatment with PARPi contributes to synthetic lethality by driving MRE11-dependent RF resection. However, there is also evidence to suggest that PARP1 itself recruits MRE11 to stalled RFs [87], and that deficiencies in PARP1 may promote chemotherapeutic resistance in BRCA2-deficient cells by limiting the access of MRE11 to single-stranded DNA at stalled RFs [118]. Moreover, simultaneous loss of both PARP1 and BRCA1 actually protects against genome instability in *Brca1^−/−^Parp1^−/−^* knockout cells [118]. Thus, the role of PARP1 at stalled RFs appears to be either protective or deleterious depending on the cellular context.

### 2.3. Clinical PARP Inhibitors

**Figure 3:**
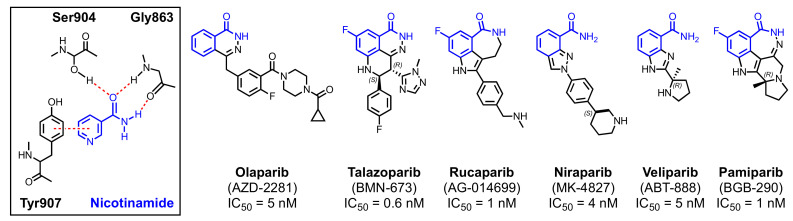
FDA-Approved and Clinically Advanced PARP Inhibitors. The panel inset depicts nicotinamide (blue) and its highly conserved interactions with PARP1-4, including hydrogen bonds with serine 904 and glycine 863 and a prominent π-stacking interaction with tyrosine 907 (using PARP1 amino acid numbers). The FDA-approved PARPi olaparib, talazoparib, rucaparib, and niraparib as well as the clinically advanced inhibitors veliparib and pamiparib are compared. The nicotinamide motif of each inhibitor is depicted in blue. The reported IC_50_ values for inhibition of purified PARP1 enzymatic activity are also provided.

All PARPi that have advanced in the clinic are structural analogs of nicotinamide (Figure 3). Each of these agents competitively inhibits the binding of endogenous NAD^+^ to the PARP1 and PARP2 active sites and thus prevents catalytic activity. Within the active site, nicotinamide makes three crucial hydrogen bond interactions with the hydroxyl group of Ser904 and the amide backbone of Gly863 [123,124]. The pyridyl ring is further positioned and stabilized by a prominent π-stacking interaction with Tyr907 (left panel in Figure 3). By design, the PARPi shown in Figure 3 recapitulate these interactions and others to enhance specificity and potency [93]. Four PARPi have been approved by the FDA: olaparib (2014), rucaparib (2016), niraparib (2017), and talazoparib (2018).

Additionally, shown in Figure 3 are the reported IC_50_ values for each PARPi with purified PARP1. It is important to note, however, that the cellular potency of these inhibitors varies far more widely than these IC_50_ values, likely reflecting differences in susceptibility of these agents to drug efflux pumps [125,126] and varied effects of the PARP1-binding protein HPF1 on the abilities of these agents to trap PARP1 on the DNA [127].

#### 2.3.1. Olaparib

Olaparib is a prototypical PARPi built upon a phthalazinone core that was identified as a moderately potent PARP1 antagonist in a medium throughput screen [128]. While early compounds in the series exhibited little efficacy in whole-cell assays, the incorporation of the pendant benzyl linker afforded potent PARP1 inhibition at both the enzyme and cellular level. This activity was further enhanced via the addition of a 1-carbonyl-1,4-diazepane moiety. Substitution of the diazacycloheptane for piperazine and N4-alkylation with cyclopropanecarbonyl improved oral bioavailability and cellular potency, respectively, yielding the final compound olaparib. In tumor cell lysates, PARP activity is inhibited >90% at 100 nM olaparib, and in colony forming assays, an EC_50_ value of ~250 nM olaparib was observed with a *BRCA1*-mutant breast cancer line incubated for 7–14 days [128]. While designed as a PARP1 inhibitor, olaparib also exhibits submicromolar potency against PARP2-4 due to the high level of similarity between catalytic domains of these members of the PARP superfamily [92,93]. Olaparib was first FDA-approved in December 2014 for advanced *BRCA*-mutant ovarian cancer.

#### 2.3.2. Talazoparib

Talazoparib is the most potent clinical PARPi described to date, with a reported EC_50_ value of 0.3 nM in killing *BRCA*-mutant MX-1 cells ex vivo when incubated for 10–12 days [129]. This potency is attributed to the high efficiency with which talazoparib traps PARP1-DNA complexes in comparison to other PARPi [130]. Talazoparib is based upon a tetrahydropyridophthalazinone core with 4-fluorphenyl and 1-methyl-1,2,4-triazol-5-yl *trans*-disubstitution at the 8- and 9-positions, respectively. Compared to the *cis*-addition counterparts, the *trans* isomers are both the thermodynamically favored reaction products and more potent PARP1 inhibitors [129]. Importantly, of the two *trans* isomers, it is the (*8S*, *9R*)-enantiomer that is the most active compound, exhibiting a >200-fold improvement in potency when resolved from the racemate. Structurally, the (*8S*, *9R*) conformation allows the fluorophenyl and 1,2,4-triazole groups to form unique π-stacking and water-mediated hydrogen bonding interactions with Tyr899 and Tyr896 of PARP1, respectively. In contrast, the conformation of the (*8R*, *9S*) enantiomer displaces the ligand within the NAD^+^ binding site, impairing the critical π-stacking interaction with Tyr907 and preventing the formation of the additional water-mediated hydrogen bond with Tyr896, thereby rationalizing the vastly different potency of the two enantiomers [129]. Talazoparib is marketed as strictly the (*8S*, *9R*)-enantiomer, though precise enantiopurity is not reported. Finally, similar to olaparib, talazoparib inhibits PARP1-4 [93]. Talazoparib was first FDA-approved in October 2018 for advanced *BRCA1/2*-mutant breast cancer.

#### 2.3.3. Rucaparib

Rucaparib is built upon a tricyclic indole scaffold with a constrained amide [131]. Similar to talazoparib, rucaparib makes additional interactions with Tyr896 that contribute to enhanced potency [132]. However, replacement of the azepino ring with a diazepino moiety in an attempt to form additional stabilizing interactions failed to produce more potent analogs [132]. Rucaparib is less selective for PARP1-4 than olaparib or talazoparib, with modest activity against PARP10 and TNKS1-2 [93]. Crystal structure comparisons suggest that this mild promiscuity may be due to the flexibility of the terminal secondary amine, which can facilitate alternate binding patterns depending on the local environment [93]. Rucaparib was first FDA-approved in December 2016 for advanced *BRCA1/2*-mutant ovarian cancer.

#### 2.3.4. Niraparib

Niraparib is based upon a fused aromatic azabicycle scaffold that, rather than covalently constraining the amide motif, relies upon an intramolecular hydrogen bond between a pyrazole nitrogen and the *anti* hydrogen of the amide [133]. Installation of a 3-phenylpiperadine moiety yielded a compound with potent activity (IC_50_ = 3 nM) against PARP1 and promising cellular activity [133]. Similar to talazoparib, separation of the two enantiomers revealed disparate properties. While the *R*- and *S*-enantiomers had similar activity against purified PARP1 (IC_50_ values of 2.4 nM and 3.2 nM, respectively), the *S*-enantiomer was an order of magnitude more potent with respect to inhibition of PARylation activity in HeLa cells (EC_50_ values of 30 nM and 4 nM, respectively) and thus became niraparib [134]. Interestingly, niraparib is highly selective for PARP1 and PARP2, as it makes additional interactions with the backbone residues Asp766 and Glu335, respectively, side chains that are not present in other PARP family members [93]. Niraparib was first FDA-approved in March 2017 for maintenance therapy of platinum-sensitive ovarian, fallopian tube, or primary peritoneal cancers.

#### 2.3.5. Veliparib

Similar to niraparib, veliparib utilizes an aromatic bicycle core (a benzimidazole) that constrains the amide group via an intermolecular hydrogen bond [135]. It also makes distinct interactions with the Glu763 and Glu335 residues of PARP1 and PARP2, respectively, enhancing its selectivity for these PARPs [93]. In fact, veliparib is the most selective inhibitor of PARP1 and PARP2 and, with IC_50_ values >100-fold lower than for other family members, is the only PARPi to meet chemical probe criteria [93]. However, despite similar inhibitory potency with respect to PARP1 activity (IC_50_ = 5 nM) and cellular PARylation (EC_50_ = 6 nM), veliparib has only modest PARP-trapping efficacy (GI_50_ = 6 µM) and is an order of magnitude less potent in its ability to sensitize cells to temozolomide [28,129]. Veliparib has recently completed phase III clinical trials in newly diagnosed ovarian cancer, early-stage triple-negative breast cancer, and both advanced squamous and non-squamous non-small cell lung cancer, but it has yet to receive FDA approval.

#### 2.3.6. Pamiparib

Pamiparib is the most recently developed PARPi. It is a pentacyclic dihydrodiazepinoindolone derivative that, similar to rucaparib, incorporates a seven-membered ring to lock the carboxamide group into the biologically active conformation [136]. Depending on the assay, the active (*R*)-enantiomer of pamiparib exhibits an IC_50_ value of 1.3–5.1 nM against PARP1, and the DNA-trapping activity is similar to that of olaparib and rucaparib [136,137]. It is also a potent inhibitor of PARP2 (IC_50_ = 0.9 nM), with modest activity against PARP3 (IC_50_ = 68 nM) [136,137]. Based on the results of a combined phase I/II trial (NCT03333915), pamiparib was recently approved in China for the treatment of relapsed/refractory germline *BRCA*-mutant ovarian, fallopian, or primary peritoneal cancer [138]. However, pamiparib has not yet been approved by the FDA, and its activity in myeloid neoplasms has not been reported.

## 3. PARP Inhibitors for the Treatment of Myeloid Neoplasms

### 3.1. Rationale for PARP Inhibition in Myeloid Neoplasms

While *BRCA1/2* mutations are uncommon in hematologic malignancies [35], clinical experience has demonstrated benefits of PARPi in cancers with HR deficiencies due to a myriad of other gene mutations [16,17,139]. Leukemia cells are characterized by a high degree of chromosomal instability that is thought to arise from faulty DNA damage repair mechanisms [44,140], including dysregulation of several genes involved in HR, such as *ATM*, *ATR*, *CHK1*, and *RAD51* [41,141]. Moreover, there is evidence to suggest that an impaired DDR contributes to arrest of myeloid blast differentiation, leukemia pathogenesis, and treatment resistance [140,142]. These considerations have led to assessment of PARP inhibition as a strategy to intervene in myeloid neoplasms, particularly those with demonstrated genomic instability and chromosomal aberrations.

### 3.2. Pre-Clinical Efficacy in Myeloid Neoplasms

AML is a heterogeneous disease with diverse underlying molecular aberrations. Therefore, it is not surprising that PARPi monotherapy has produced mixed results when tested both in vitro and in vivo. For example, when olaparib was tested against a panel of AML cell lines (including HL-60, NB4, OCI-AML2, and OCI-AML3) and primary patient samples, a response was seen in most (88%) but not all cases, and the degree of susceptibility varied considerably [40,143]. In other reports, however, olaparib monotherapy had no effect in the same HL-60 cell line [144]. Primary AML samples that responded to olaparib were found to have reduced levels of *BRCA1* expression, while those that were resistant overexpressed *PARP1* [40,145]. Moreover, of all primary samples in the panel, the highest sensitivity was seen in an AML harboring a deletion at chromosome 11q23 in the region of the *MRE11A*, *ATM*, and *H2AX* genes [40]. In contrast, 11q23 rearrangements with *MLL* maintain HR proficiency, and AML blasts driven by these *MLL* fusions are insensitive to PARP inhibition [38]. These results indicate that PARPi sensitivity depends on the molecular alterations driving the leukemia.

Subsequent work has characterized the mixed effects of PARP inhibition in several subtypes of leukemia and myeloproliferative disorders (Table 1). While *BRCA* mutations are rare in myeloid neoplasms, several genetic anomalies have been associated with functional HRD, producing a similar mutator phenotype in these malignancies. A gene expression and mutation analysis (using RT-qPCR, microarray analysis, and flow cytometry) of a panel of primary CML and AML samples directly identified functional deficits in BRCA- and DNA-PK-mediated DNA repair pathways and accurately predicted sensitivity to PARPi therapy in these samples [146]. In this analysis, leukemias expressing the fusion proteins BCR-ABL1 and RUNX1-RUX1T1 were most responsive, a finding that has been recapitulated in separate studies of these subtypes [38,147,148]. HRD and PARPi sensitivity has also been reported for leukemias harboring *IDH1/2* mutations [149,150,151], *PML-RARα* translocations [38,152], and cohesin complex aberrations [153]. These preclinical studies indicate that PARP inhibition may be most efficacious in myeloid malignancies with underlying HR deficiencies.

PARPi sensitivity can also be dramatically enhanced via combination therapy (Table 2). These combinations work through several mechanisms. For example, temozolomide increases N^7^-methylguanine, which requires repair through the PARP1-dependent BER pathway [154]. DNA topoisomerase I (TOP1) poisons such as camptothecin or topotecan induce stalling of RFs, which depend in part on PARP1 for resolution [155]. Hypomethylating agents result in formation of DNA methyltransferase (DNMT)-DNA covalent adducts, which might require PARP1-dependent processes for their removal [156]. In addition, hypomethylating agents have been shown to downregulate *RAD51*, *BRCA1*, *BCRA2*, *FEN1*, and *FANCD2* to induce HRD as well as sensitivity to PARP inhibition in several AML cell lines and primary samples [157]. Accordingly, combination therapy with decitabine and PARPi was shown to significantly reduce colony formation in primary AML samples and prolong survival in murine AML xenograft models [156]. Induced HRD is also achieved in *FLT3-ITD^+^*, *BCR-ABL1^+^*, and *JAK2*-mutant neoplasms after treatment with quizartinib [158], imatinib [146,159], and ruxolitinib [160], respectively, leading to considerable synergy with PARPi across various models in vitro and in vivo. Conversely, PARPi treatment also increases susceptibility to other interventions. For instance, PARP inhibition upregulates the death receptors TNFRSF6 and TNFRSF10B, with the latter conferring increased sensitivity to TNF-related apoptosis-inducing ligand (TRAIL) [161]. Ultimately, rational therapeutic combinations have led to increased synergy with and sensitivity to PARPi across several molecular subsets of myeloid neoplasms, prompting early efforts at clinical translation.

**Table 1:** Preclinical Results of PARP Inhibitor Monotherapy in Defined Molecular Subtypes of Myeloid Neoplasms.

Disease	Genotype(s)	Phenotype	Results of PARPi Monotherapy	Ref(s)
AML	*FLT3-ITD* mutant	Upregulation of RAD51 via STAT5 activation. Rapid depletion of γH2AX with highly active DSB repair.	Modest anti-leukemic activity seen with PARPi monotherapy in cell lines. Reduction in AML-initiating *FLT3-ITD+* cells and clonogenic cells in bone marrow under hypoxic conditions. No significant reduction in leukemic burden or prolongation of survival in primary *FLT3-ITD+* AML murine xenografts.	[158,162]
AML	*IDH1/2* mutant	Increased 2HG inhibits KDM4A/B, ALKBH, ATR, and ATM to induce HRD and DSB persistence.	Primary *IDH1/2*-mutant AML cells possessed a 2HG-dependent DSB repair defect that conferred sensitivity to PARPi in vitro; sensitivity was reversed with IDH1/2 inhibitors.	[149,150,151]
AML	*RUNX1-* *RUNX1T1* *(AML1-ETO)* positive	Downregulation of DNA repair genes, including *BRCA2.* High mutation frequency with mutator phenotype. Aberrant *TET1* expression and DNA methylation.	Reduced colony-forming potential in RUNX1-RUNX1T1 transformed primary cells and patient-derived cell-lines. Prolonged survival in RUNX1-RUNX1T1 AML xenograft model. DNA damage-induced differentiation of PML-RARα transformed leukemic blasts.	[38,42,148,163]
AML	Cohesin (*STAG2*) mutant	High dependency on DDR pathways. Increased replication fork stalling.	AML (including *STAG2*-mutant) cell lines were sensitive to PARPi both in vitro and in vivo (xenograft model). Primary *STAG2*-mutant AML samples exhibited dose-dependent sensitivity to PARPi. PARPi depleted cohesin-mutant clones in a *Tet2/Stag2*-mutant murine model of MDS/AML.	[153]
APL	*PML-RARα* positive	Reduced *MSH6*, *MLH1*, *BRCA1*, and *RAD51* expression. Repression of *CHEK1*, *CHK2*, and several BER genes induces a mutator phenotype.	Reduced colony-forming potential in *PML-RARα* transformed primary cells and patient-derived cell-lines. Suppressed disease onset in an ATRA-resistant APL xenograft model. DNA damage-induced differentiation of *PML-RARα* transformed leukemic blasts.	[38,39,152,164]
CML	*BCR-ABL* positive	Reduced translation of *BRCA1* mRNA. Functional BRCA1 deficiency. HR downregulation and accumulation of DSBs.	Increased DSBs and reduced clonogenic potential of imatinib-refractory CML cell lines and primary samples, including under hypoxic conditions mimicking the bone marrow microenvironment. Eliminated quiescent cells in an inducible mouse model of chronic-phase CML. Reduced leukemic burden up to 10-fold in a BCR-ABL1^+^ leukemia xenograft model.	[146,147,159]
MLL	*MLL-AF9*	High burden of oxidative DNA damage. Increased PARP1 expression and acetylation.	*MLL-AF9* transformed murine bone marrow cells were only modestly sensitive to PARPi monotherapy. RUNX1-RUNX1T1-positive murine cells were highly sensitive to PARPi. Reduced the number of leukemic stem cells in primary human AML (MLL-AF9^+^) samples in vitro. PARPi and cytotoxic drugs (doxorubicin and cytarabine) exert additive anti-MLL-AF9 leukemia effects in mice. No significant reduction in leukemic burden was seen in a syngeneic mouse model of MLL-AF9^+^ leukemia (except when PARP inhibition was combined with cytotoxic drugs). *MLL-AF9*-transformed cells were resistant to olaparib monotherapy. No significant effect of olaparib on mice transplanted with wild-type MLL-AF9 leukemic cells. *Hoxa9*-deficient MLL-AF9 cells were highly sensitive to PARPi.	[38,165,166,167]
MPN	JAK2 (V617F) MPL (W515L) CALR (del52) positive	Reduced formation of RAD51 foci. Modest down-regulation of BRCA1/2. Accumulation of ROS-induced DSBs.	Modest in vitro sensitivity across several MPN cell lines, though sensitivity of primary MPN samples was variable. Primary MPN cells exhibited reduced colony formation in vitro after PARPi treatment. Veliparib monotherapy did not significantly prolong survival in a murine xenograft model.	[160,168,169]

Abbreviations: 2HG, 2-hydroxyglutarate; ATO, arsenic trioxide; DDR, DNA damage response; MDS, myelodysplastic syndrome; PARPi, PARP inhibitor; Ref, reference.

### 3.3. Clinical Efficacy in Myeloid Neoplasms

Several early phase clinical trials have evaluated PARPi for the treatment of hematologic malignancies (Table 3). One of the first reported phase I trials assessed the use of single-agent talazoparib in two small cohorts of patients, including those with AML or myelodysplastic syndrome (MDS) (*n* = 25) or those with chronic lymphocytic leukemia (CLL) or mantle cell lymphoma (MCL) (*n* = 8) [180]. All patients had relapsed or refractory disease, with a median of either 3 or 6 prior treatment regimens, respectively. Talazoparib was administered on a continuous daily schedule in 21-day cycles at escalating dose levels, ranging from 100–2000 µg/day. Dose-limiting toxicities included severe neutropenia in 2 of 5 patients at a dose of 900 µg/day and neutropenic fever or sepsis in 2 of 4 patients at 2000 µg/day. While no objective responses were seen, stable disease was reported in 13 of 25 patients (52%) in the AML/MDS arm and in 5 of 8 patients (63%) in the CLL/MCL arm. One patient with MDS received 24 cycles of talazoparib over 484 days and became independent of red blood cell (RBC) transfusions. Otherwise, the duration of disease stability or follow up of the other patients was not specified.

While single-agent PARP inhibition was relatively well tolerated, the modest clinical efficacy of this strategy in myeloid neoplasms prompted investigation of combination therapies. Veliparib was combined with temozolomide in a phase I study of 48 patients with relapsed/refractory AML [181]. The median age was 69 years (range 20–88 years) with a median of two prior therapies (range 0–6), including nine patients who had previously undergone allogeneic hematopoietic stem cell transplantation (allo-HSCT). Patients received escalating doses of veliparib (40–200 mg twice daily) coupled with a stable dose of temozolomide (150–200 mg/m^2^) in 28-day cycles. The maximum tolerated dose (MTD) of veliparib was 150 mg twice daily, with two of four patients (50%) at the 200 mg twice daily dose experiencing grade three oropharyngeal mucositis/esophagitis lasting >7 days. Infections (40%) and febrile neutropenia (25%) were also observed, with increasing frequency at higher doses. A complete response (CR) was attained in 8 of 48 patients (17%), with seven of these patients achieving CR after the first cycle. An additional seven patients experienced hematologic improvement (HI) or disease stabilization. The median overall survival (OS) for all patients was 5.3 months. Patients who achieved CR had a median OS of 20 months, while those who achieved HI or stable disease experienced a median OS of 9.4 months. In the pharmacodynamic analysis, escalating doses of veliparib were associated with dose-proportional inhibition of baseline pADPr polymer content. In addition, a veliparib-induced increase in phosphorylated H2AX was observed in the CD34^+^ cells of responders. Moreover, three of four patients with *MGMT* promoter methylation achieved CR, suggesting that methylated *MGMT* may be a biomarker for sensitivity to this regimen.

Veliparib was also assessed in combination with topotecan and carboplatin in a phase I study of 99 patients with relapsed/refractory AML, chronic myelomonocytic leukemia (CMML) or an aggressive MPN [182]. The median age for all patients was 56 years (range 25–76 years) with a median of two prior therapies (range 0–4), including 16 patients with prior allo-HSCT. Escalating doses of veliparib (10–100 mg twice daily) were administered alongside standard doses of topotecan (1.0–1.3 mg/m^2^/day) and carboplatin (120–150 mg/m^2^/day) in 21-day cycles. The MTD of veliparib was 80 mg twice daily for up to 21 days, with two of four patients (50%) at the 90 mg twice-daily dose experiencing grade ≥3 mucositis. Clinical responses were observed across a wide range of doses, including 14 CRs (14%), 11 CRs with incomplete count recovery (CRi, 11%), and 8 partial responses (PRs, 8%) for an overall response rate of 33%. Among patients with de novo AML, the overall response rate was 25% (19/77). However, responses were seen in 64% (14/22) of patients with aggressive MPNs, CMML, or secondary AML, of whom 11 subsequently proceeded to allo-HSCT with donor cell engraftment. While data for all patients were not reported, the median OS was 15.3 months for responders and 4.2 months for non-responders. As in the study by Gojo et al., decreased pADPr content and increased H2AX phosphorylation were observed in circulating CD34^+^ blasts at higher drug doses. Furthermore, impaired monoubiquitination of FANCD2 was detected in 28 of 49 tested samples (57%) and was associated with a modest prolongation of survival (median 6.1 months versus 4.8 months and one-year survival 39% versus 5%). A phase II trial of the topotecan/carboplatin/veliparib combination is ongoing.

These early trials were conducted in unselected patients with myeloid neoplasms. As discussed above, AML subtypes with molecular deficits that contribute to HR deficiency may have increased sensitivity to PARP inhibition [141,184]. New trials assessing PARPi in these molecular subsets of AML have begun (Table 4). Specifically, olaparib is being evaluated in a phase II study of *IDH1/2* mutant AML (NCT03953898), while talazoparib is being assessed in a phase I study of cohesin-mutant AML (NCT03974217).

### 3.4. Biomarkers of PARPi Sensitivity

The varied results of both preclinical and clinical studies highlight the need to identify reliable biomarkers of PARPi sensitivity. Early siRNA screens in breast cancer cell lines revealed that deficiencies in several DNA-repair genes other than *BRCA1* and *BRCA2*, including *ATM*, *CHEK1*, *CDK5*, *XRCC1*, *LIG1*, *PCNA*, *XAB2*, and *DDB1*, may confer susceptibility to PARP inhibition [185,186]. Moreover, the disease-defining cytogenetics that confer HRD in myeloid malignancies—such as translocations of *BCR-ABL* [147], *RUNX1-RUNX1T1* [146,148], and *PML-RARα* [38] or mutations in *IDH1/2* [149,150] or cohesin complex genes [153]—may predict PARPi sensitivity in these disease subtypes. However, additional clinical data are needed to determine whether PARP inhibition is reliably efficacious in these settings.

Clinical trials of PARPi in myeloid neoplasms have examined biomarkers in cytogenetically diverse and unselected populations. The study by Gojo et al. assessed whether methylation of the *MGMT* (O^6^-methylguanine-DNA methyltransferase) promoter was associated with response to therapy with temozolomide plus veliparib in relapsed/refractory myeloid leukemias and myeloproliferative neoplasms [181]. When the *MGMT* promoter is methylated, MGMT protein is not expressed, and cells need to rely on alternative, PARP1-dependent mechanisms to remove O^6^-methylguanine that is formed upon temozolomide treatment. While three of four patients with *MGMT* hypermethylation achieved CR, responses were also seen in patients without promoter hypermethylation. All 19 cases of AML in this trial were noted to have impaired FANCD2 ubiquitination, consistent with the notion that defects in the Fanconi anemia pathway are common in poor-risk myeloid malignancies. Pratz et al. assessed this further in their trial of veliparib plus topotecan and carboplatin in relapsed/refractory AML, MPN, or CMML [182]. Impaired FANCD2 monoubiquitination was detected in 28 of 49 samples (57%) and was associated with a modest survival benefit (median 6.1 vs. 4.8 months, *p* = 0.034). Clearly, additional work is needed to delineate clinically relevant biomarkers predictive of PARPi sensitivity in hematologic malignancies.

### 3.5. Mechanisms of PARP Inhibitor Resistance

Mechanisms of PARPi resistance specific to myeloid neoplasms are understudied [187]. *BRCA* reversion mutations that restore the open reading frame or reversal of promoter hypermethylation are well-known to induce PARPi resistance in breast and ovarian cancers in the preclinical setting [188,189,190] and in the clinic [191,192,193,194,195]. In addition, PARP1 point mutations, especially those within the DNA-binding zinc finger domains, can confer PARPi resistance by altering PARP1 trapping, and at least one such mutation has been detected in a clinical sample [196].

Mutations that impair the DNA end resection necessary for NHEJ have also been implicated in PARPi resistance. Loss of 53BP1 alleviates the PARPi hypersensitivity of *BRCA*-mutant cells by promoting ATM-dependent processing of damaged DNA, thereby producing ssDNA suitable for high-fidelity repair via HR [197,198]. The nuclease Artemis is a PTIP-binding protein that acts downstream of 53BP1 and is a major effector of the NHEJ pathway. As expected, loss of Artemis confers PARPi resistance in BRCA1-deficient cells as well [199].

Enhanced replication fork stability may also contribute to PARPi resistance. As discussed above, the MRE11 exonuclease is recruited to stalled replication forks, where it contributes to end resection until the DNA lesion has been removed. MRE11 recruitment is enhanced by PTIP, and loss of PTIP has been shown to protect replication forks from extensive degradation in BRCA-deficient cells [118]. Such replication fork protection confers chemoresistance to PARPi by stabilizing the replication fork even in the absence of both BRCA2 and PARP1 activity.

Finally, the regulatory microRNA miR-181a has been shown to be downregulated in *MLL*-rearranged leukemias, where it contributes to impaired acetylation of PARP1 [200]. Forced overexpression of miR-181a in *MLL*-rearranged cell lines (THP-1 and SHI-1) restored sensitivity to PARP inhibition. Additional work is needed to identify which resistance mechanisms are most relevant in myeloid neoplasms and whether additional methods unique to these malignancies exist.

### 3.6. Challenges and Future Directions in Development of PARP Inhibitors for Myeloid Neoplasms

As the armamentarium of therapeutics in myeloid malignancies continues to expand, exploring the potential role of PARPi as part of combination therapies may offer options for subsets of patients with myeloid neoplasms for whom specific or targeted inhibitors have not yet been developed. As an example, atypical CML is a rare *BCR-ABL1*-negative hematologic malignancy in which PARP1 overexpression is associated with a poorer prognosis [201,202]. In other contexts, combinations with ruxolitinib in MPN or FLT3 inhibitors in *FLT3-ITD*-positive AML may be of interest, as these subsets of myeloid neoplasms are characterized by increased genomic instability [203,204]. Likewise, combining PARPi with hypomethylating agents (decitabine or azacitidine) could be promising in the treatment of MDS and AML [156,166].

A variety of agents that induce or prolong DNA damage are known to induce senescence as an alternative outcome to target cell apoptosis [205]. Recent studies have demonstrated that BH3 mimetics such as navitoclax or venetoclax, which selectively inhibit certain anti-apoptotic BCL2 family members, can selectively kill senescent cells [206,207,208]. Accordingly, there is reason to believe that combining PARPi with BH3 mimetics may capitalize on PARPi-induced senescence and subsequently enhance the killing of neoplastic cells. Such combinations may, however, be limited by the propensity of PARPi and BH3 mimetics to induce bone marrow toxicity even as monotherapy, let alone in combination [209]. A potential strategy to mitigate this toxicity might involve administering PARPi therapy followed by BH3 mimetics [209]. Along those same lines, while hypomethylating agents and the BCL2 inhibitor venetoclax demonstrate therapeutic synergy in the clinic [210], the safety and tolerability of adding an additional agent such as a PARPi to this doublet remain to be established. Once again, there is concern that toxicities, especially prolonged myelosuppression and gut toxicity, might be limiting with currently established doses and regimens.

Emerging data have highlighted the role of PARP enzymes in epigenetic regulation, providing another opportunity for future exploration of PARPi in the treatment of hematological malignancies. For example, PARP1 activity upregulates the expression of ten-eleven translocation methylcytosine dioxygenase 1 (TET1) [211] and stimulates TET1 activity in a context-dependent manner [212]. In T cell acute lymphoblastic leukemia (T-ALL), where both PARP1 and TET1 are highly expressed, PARP inhibition with olaparib reduces TET1 expression and antagonizes T-ALL cell growth [213]. It remains to be seen whether a similar association between PARP1 and TET1 exists in myeloid neoplasms. Conversely, somatic TET2 deficiency is a common feature of hematologic malignancies and is associated with downregulation of BRCA1 and LIG4, leading to impaired HR and NHEJ, respectively [214]. Thus, TET2-deficient cells are increasingly reliant on the PARP1-mediated alt-EJ DNA repair pathway, conferring sensitivity to PARPi therapy both in vitro and in vivo [214,215]. In this manner, *TET2* mutations may serve as a biomarker for PARPi sensitivity in epigenetically dysregulated malignancies.

Finally, studies of PARPi in patients with solid tumors have identified fatigue and nausea as troubling side effects during long-term treatment. As studies move forward in myeloid malignancies, the effects of PARPi therapy on quality of life and patient-reported outcomes (PRO) should be incorporated in a prospective fashion into trials testing those agents as monotherapy and in combination [216]. Because the completion of PRO instruments may be burdensome to patients, investigators should consider limiting those assessments to questions that inform adherence to therapy, improve disease-specific symptoms, and focus on common symptoms seen with these agents in the solid tumor setting [217].

## 4. Myeloid Neoplasms Emerging with PARP Inhibitor Therapy

### 4.1. Recognition of PARP Inhibitor Related Myeloid Neoplasms

In addition to serving as potential therapeutic agents for myeloid neoplasms, PARPi are also emerging as a cause of these disorders [19]. Therapy-related myeloid neoplasms, which include therapy-related AML, MDS, and MDS/MPN overlap, are typically encountered as a late complication of chemotherapy or radiation therapy [218]. Different subtypes of therapy-related myeloid neoplasms have varying latency periods from the time of exposure to chemotherapy or radiation therapy. For instance, alkylating agents and radiation therapy are associated with myeloid neoplasms that often present as MDS with subsequent progression to AML and are characterized by deletions of chromosome five or seven, changes that are associated with an unfavorable response to therapy [219]. Topoisomerase II inhibitors are associated with another subtype of therapy-related myeloid neoplasms that emerge within 1–2 years of exposure, present as acute leukemia without antecedent MDS [220], are associated with translocations involving *MLL* or *RUNX1* and have higher rates of response to leukemia-directed therapy [219].

Several processes might contribute to the development of therapy-related myeloid neoplasms, including therapy-induced increases in genomic instability with subsequent accumulation of aberrations [221] and the selection of a founder population of hematopoietic stem cells with predisposing clonal hematopoiesis (CH) mutations, such as *TP53* mutations [219,222]. In this context, CH refers to the clonal expansion of a subpopulation of hematopoietic stem cells with a preexisting somatic mutation in the absence of overt signs of MDS or AML [223]. While older age is an established risk factor for CH, exposure to DNA-damaging modalities, including the chemotherapy that often precedes treatment with PARPi, may facilitate the emergence of clones exhibiting improved fitness in the face of DNA damage [224]. Moreover, when compared to de novo myeloid malignancies, therapy-related myeloid neoplasms are more likely to harbor mutations in components of the DDR pathway, such as *TP53* and *PPM1D* [225,226,227]. Similar to chemotherapy and radiation therapy, PARPi therapy may select for and promote the expansion of hematopoietic stem cell clones with mutations in *TP53* and *PPM1D* [228,229,230].

The association of PARPi therapy with the emergence of myeloid neoplasms, specifically MDS and AML, has been examined since the early clinical studies of PARPi. PARPi therapy-related myeloid neoplasms have been reported to have an incidence of 1–3% [16,19,191,231,232]. While the individual clinical trials studying PARPi, including SOLO2 [232], did not show a statistically significant difference in the rate of myeloid neoplasms in the PARPi group when compared with the placebo group, those studies were underpowered to examine this particular adverse event. As a result, the relatively higher rates of myeloid neoplasms observed in those trials were initially thought to be related to platinum-based therapy. A subsequent meta-analysis, however, not only confirmed the increased risk of myeloid neoplasms with increased platinum therapy, but also showed that PARPi therapy is associated with a two- to three-fold increased risk of AML and MDS relative to patients with the same diagnoses treated with the same therapy but without the PARPi [19].

This possible risk of MDS and AML becomes highly relevant as the use of PARPi expands to arenas where cancer is curable [233,234]. For instance, the growing use of PARPi for prolonged maintenance therapy following first-line platinum-based chemotherapy in ovarian cancer [235,236] highlights the need of better understanding this risk, especially when considering that therapy-related myeloid neoplasms are associated with high morbidity and mortality [237]. In this context, there are several questions regarding the pathogenesis of PARPi-emergent myeloid neoplasms that must be answered to better inform clinical decisions (Box 1).

Box 1 **Outstanding Questions About PARPi-Emergent Myeloid Neoplasms That Need to be Answered.**

1. Is there a subset of patients who are at a particularly high risk of developing therapy-related MDS or AML while receiving treatment with a PARPi?
2. If so, how can we identify this group of high-risk patients to better stratify the risks and benefits of PARPi therapy?
3. Do germline mutations in *BRCA1*, *BRCA2*, *BARD1*, *RAD51*, *TP53*, or *PALB2*—which are commonly encountered in patients with ovarian or breast cancer—confound the picture by increasing the risk of therapy-related MDS and AML?
4. Is the risk of therapy-related myeloid neoplasms cumulative with continued PARPi therapy?
5. What is the contribution of other DNA-damaging modalities—including conventional chemotherapy and radiation therapy—to the emergence of therapy-related myeloid neoplasms?

### 4.2. Epidemiology and Characteristics of PARPi-Related Myeloid Neoplasms

In an attempt to answer these questions, a recent report identified 11 patients with MDS and 9 patients with AML following PARPi therapy with predominantly olaparib (Table 5) [238]. These therapy-related myeloid neoplasms were diagnosed at a median of 2 years after initiation of PARPi treatment [238]. Unfavorable cytogenetics, particularly complex karyotypes, were found in the overwhelming majority of cases [238]. Mutations in DDR genes were detected by targeted next generation sequencing (NGS) in 83% of the cases [238]. CH was more common in patients with ovarian cancer on maintenance PARPi therapy when compared to those not receiving PARPi maintenance (78% vs. 39%, respectively) and showed expansion in paired specimens pre- and post-therapy [238]. Along the same lines, mutations in DDR genes, including *TP53* and *PPMI1D*, were more common in patients receiving maintenance PARPi than in those not receiving maintenance (67% vs. 17%) [238].

Additional insight comes from a meta-analysis by Morice et al. that captured data from randomized controlled trials with PARPi in different solid tumor types [19]. In the 18 placebo-controlled trials examined, PARPi therapy was associated with a significantly higher risk of therapy-related AML or MDS, with a Peto odds ratio of 2.63 (95% CI 1.13–6.14) [119]. While those findings present a risk that warrants well-designed follow-up investigations, it is noteworthy that the incidence of MDS and AML was relatively low at 0.73% in the pooled PARPi group versus 0.47% across placebo groups, with a median follow-up ranging between 8.2 and 78 months [19]. In an independent analysis nested within the same publication, the authors use VigiBase, a pharmacovigilance database, to report 178 cases of MDS and AML with various PARPi (niraparib, olaparib, rucaparib, talazoparib, and veliparib), with a median PARPi treatment duration of 9.8 months (range: 0.2–66.8 months) (Table 5) [19]. In cases where latency data were available (58 of the 178 cases), the median time from first exposure to PARPi to diagnosis of MDS or AML was 17.8 months (range: 0.6–66.8 months) [19]. Data about previous lines of therapy prior to PARPi exposure were available for only 13 patients, all of whom had received platinum- or taxane-based chemotherapy [19].

In a more recent retrospective case–control analysis of patients with ovarian cancer enrolled on the ARIEL2 and ARIEL3 ovarian cancer studies, pre-existing *TP53* CH mutations were found to be significantly associated with the development of therapy-related myeloid neoplasms after exposure to rucaparib [239]. This analysis, which was based on targeted NGS of peripheral blood cell specimens from 20 patients who developed therapy-related myeloid neoplasms and 44 patients who did not, demonstrated that the prevalence of preexisting CH variants in *TP53* at a variant allele frequency of ≥1% was significantly higher at PARPi treatment initiation in peripheral blood cells from patients who ultimately developed therapy-related myeloid neoplasms compared to controls who did not (9 (45.0%) of 20 cases vs. 6 (13.6%) of 44 controls, OR 5.2 (95% CI 1.6–16.0, *p* = 0.009)). In contrast, other CH variants were not enriched in the patients who went on to develop therapy-related myeloid neoplasms [239].

Considering the entire 1052-patient cohort from ARIEL2 and ARIEL3, Kwan et al. also explored the association between the presence of HR gene alterations and development of PARPi-related myeloid neoplasms [239]. While the prevalence of therapy-related myeloid neoplasms was higher in patients with ovarian cancer that harbored a deleterious mutation in *BRCA1*, *BRCA2*, *RAD51C,* or *RAD51D*—four genes that are commonly mutated in ovarian cancer—at 15 of 369 (4.1%) for those with mutation-containing cancers compared to 7 of 683 (1.0%) for those without mutations, the incidence was indistinguishable in patients with germline vs. somatic mutations in these genes. Instead, patients harboring ovarian cancers with HR mutations appeared to receive significantly more courses of chemotherapy, especially platinum-containing therapy, than patients with HR proficient cancers [239]. These observations provide additional assurance that patients with germline HR gene mutations are not automatically at increased risk of developing therapy-related myeloid neoplasms.

There are several challenges to consider as studies of myeloid neoplasms in the setting of PARPi therapy continue. Because PARPi provide a survival benefit (compared with a placebo), the competitive bias between death from the primary malignancy and potential therapy-related myeloid neoplasms must be carefully considered. This potential competitive bias is underscored by (i) the notion that the evolution of CH into MDS/AML is a process that may take years and (ii) the shorter durations of follow-up limiting the rates of detection of secondary myeloid neoplasms in patients who prematurely succumbed to their solid malignancy in placebo arms. Again, this highlights the importance of factoring the clinical scenario and indication into the risk and benefit assessment of PARPi therapy, with approaches adapted to disease prognosis and therapy goals in the metastatic or maintenance settings [236].

### 4.3. Reconciling the Contradictory Effects of PARP Inhibitors

The paradoxical finding of early treatment benefit followed by late risk of therapy-related myeloid neoplasms with PARPi continues to be vexing. As the role of PARPi is being explored in clinical trials for AML subtypes (*RUNX1-RUNX1T1* and *PML-RARα* fusions, *FLT3-* and *IDH1*-mutated), a shorter course of therapy with those agents to achieve synergy with chemotherapy in the upfront treatment of leukemia may have a different risk profile than prolonged use as a solid tumor maintenance therapy. As PARPi are moving up to earlier lines of therapy in many cancer types, it has become crucial to study CH evolution during PARPi exposure. These efforts may help discern whether the increased risk of therapy-related myeloid neoplasms would be mitigated by a lower baseline incidence of CH in patients who are not as heavily exposed to chemotherapy at the time they receive PARPi treatment.

Two priorities in solid malignancies are (i) better identifying patients who are at increased risk of developing therapy-related myeloid neoplasms based on the baseline CH landscape and (ii) better stratifying patients who would benefit most from PARPi therapy. In view of the emergence of therapy-related myeloid neoplasms as an important toxicity in patients receiving PARPi treatment, a third priority is identifying agents that might be effective in treating these PARPi-emergent myeloid neoplasms. These efforts could reduce the incidence of secondary malignancies as well as better inform the design of effective regimens for PARPi-related myeloid neoplasms.

For de novo leukemia therapy, clarity is also needed regarding the subgroups of patients who could potentially benefit from the addition of PARPi to current backbone regimens. If signals of benefit are seen, sequential therapy with PARPi following chemotherapy may offer a reasonable balance between the risks and benefits of those combinations.

## 5. Conclusions

The many facets of PARPi in cancer therapy continue to unfold. While promising results have emerged from studies examining PARPi (specifically olaparib, talazoparib, and veliparib) as single agents or in combination with chemotherapy in hematologic malignancies, further insight regarding the ideal therapeutic niche for these agents in myeloid neoplasms is still needed. In the meantime, focusing investigational efforts on identifying the most effective drug combinations and sequences may help further shape the role of PARPi in treating myeloid diseases. To this end, consideration must be given to both cytotoxicity in neoplastic cells as well as side effects in normal tissues, realizing that murine models might not be ideal because their intrinsic expression of drug exporters [240,241] might lead to underestimation of normal tissue toxicities of various drugs and combinations [242]. At present, the AML subtypes most likely to benefit from these endeavors are those harboring DDR pathway deficits as a consequence of *RUNX1-RUNX1T1* or *PML-RARα* fusions as well as *FLT3* or *IDH1* mutations. In parallel, a deeper understanding of the risk of therapy-related myeloid neoplasms with PARPi is a priority, as clinicians continue to counsel patients about the risks and benefits of these agents for the treatment—and possibly prevention—of ovarian cancer, breast cancer, and other solid tumors. While the incidence of therapy-related myeloid neoplasms after PARPi remains low, clinicians prescribing PARPi should remain vigilant about this possible complication. Raising awareness about the exciting role of PARPi and their potential complications has become increasingly important as these agents continue to be employed in more settings, for broader indications, in earlier lines, and for more patients.

## Figures and Tables

**Table 2:** Pre-Clinical Results of PARP Inhibitor Combination Therapy in Myeloid Neoplasms.

Class	Agent(s)	PARPi(s)	Mechanism(s)	Results of Combination Therapy	Ref(s)
Alkylating agents	Temozolomide Busulfan	Olaparib Veliparib	Temozolomide induced abasic sites and resultant SSBs. Busulfan stalled replication forks through DNA strand crosslinking. Combination with olaparib, but not veliparib, significantly increased PARP trapping.	PARPi showed synergy with temozolomide (CI < 0.3) and busulfan (CI 0.40–0.55) in vitro. With temozolomide, olaparib was >100-fold more potent than veliparib due to enhanced PARP trapping with olaparib. Busulfan plus veliparib was associated with activation of the ATR-Chk1 pathway and G2/M arrest in MPN cell lines, and modestly prolonged survival in a murine xenograft model of MPN-AML.	[169,170]
Conventional chemotherapy	Doxorubicin Daunorubicin Cytarabine 5-Fluorouracil	Olaparib Talazoparib Rucaparib	Increased abundance and phosphorylation of H2AX and CHK1. Accumulation of oxidative DNA damage. Suppression of *ATM*.	Increased PARPi sensitivity in vitro with accumulation of DNA damage, replication arrest, and apoptosis. Synergistic cytotoxicity against primary IDH1/2-mutant AML cells associated with *ATM* suppression. Rucaparib cooperates with 5-FU to accumulate DSBs in vitro and significantly enhance cytotoxicity in a syngeneic murine model of AML. Olaparib potentiates anti-leukemogenic activity of conventional chemotherapy in MLL.	[150,165,166,171]
Topoisomerase poisons	Camptothecin Etoposide	Olaparib Veliparib	Camptothecin-induced DNA lesions induce replication fork stalling, which depend in part on PARP1 for restart.	PARPi treatment was synergistic with camptothecin (CI < 0.3) in vitro; no increase in PARP/DNA complexes was detected using an insensitive assay, but genetic studies suggest a key role for PARP trapping. No synergy was seen with PARPi plus etoposide.	[104,170,172]
DNMT inhibitors	Decitabine Azacitidine	Olaparib Talazoparib	Downregulation of *RAD51*, *BRCA1/2*, *FEN1*, and *FANCD2* leads to HRD. Trapped both DNMT and PARP1 at sites of DNA damage. Repair of decitabine-induced DNA lesions is mediated by BER and requires XRCC1, recruitment of which is impaired by PARPi.	Decitabine plus olaparib was synthetically lethal in a large panel of AML cell lines, with synergy driven by PARPi-mediated inhibition of XRCC1 recruitment. DNMTi treatment induced HRD in most primary AML samples, and combination therapy (decitabine + talazoparib) significantly reduced subsequent colony formation. Combination therapy reduced leukemic burden and prolonged survival in murine AML xenografts. Synergistic antiproliferative effects against ATO-sensitive and ATO-resistant APL cell lines, with PARPi and demethylating agents (azacitidine, decitabine, and ascorbate). Synergistic cytotoxic and differentiating effects on primary MDS cells grown on culture.	[143,156,157,164,173,174]
HDAC inhibitors	Entinostat Trichostatin A Apcidin	PJ34 EB47 KU-0058948 Talazoparib	Induced DNA damage, phosphorylation of H2AX and ATM, and ultimately apoptosis. Promoted PARP trapping and impaired NHEJ via differential acetylation of Ku70/80.	HDAC inhibition enhanced PARP trapping, and co-treatment with a PARPi significantly increased apoptosis in AML cell lines. Synergistic cytotoxicity was seen with MS275 + PARPi combination therapy in select AML cell lines.	[143,175,176]
JAK2 inhibitors	Ruxolitinib	Olaparib Talazoparib	Impaired BRCA-mediated HR and DNA-PK-mediated NHEJ, thereby increasing sensitivity to PARP inhibition.	Ruxolitinib enhanced PARPi sensitivity in both MPN cell lines and primary samples with synergistic cytotoxicity in vitro. The combination of ruxolitinib, hydroxyurea, and talazoparib provided significantly greater cytoreduction than mono-/doublet therapy in both a murine MPN model and primary MPN xenograft model.	[160]
BCR-ABL inhibitors	Imatinib	Talazoparib	Downregulation of RAD51 and LIG4 to impair HR and NHEJ, respectively	Induction of DSBs and reduced clonogenic potential of imatinib-refractory CML cell lines and primary samples. Reduction in LSC-enriched quiescent cells in an inducible mouse model of chronic-phase CML. Extended disease latency in both primary and secondary recipient mice in a primary xenograft model. Synergistic 40-fold reduction in disease burden in a BCR-ABL1^+^ leukemia xenograft model.	[146,159]
FLT3-ITD inhibitors	Quizartinib	Olaparib Talazoparib Veliparib	Downregulation of BRCA1/2, PALB2, RAD51, and LIG4 impairs HR and NHEJ to induce HRD. Combination therapy caused accumulation of lethal DSBs. PARPi destabilize STAT5 to reduce aberrant FLT3-ITD signaling	Combination therapy exhibited synergistic activity against proliferating and quiescent leukemic stem/progenitor cells, eliminating both from primary AML samples. Combination therapy reduced leukemic burden in primary AML xenograft mice and prolonged survival in secondary recipients. PARPi and TKI combination therapy exhibited synergistic cytotoxicity in both TKI-sensitive and TKI-resistant AML cell lines.	[158,177]
WEE1 inhibitors	AZD1775	Olaparib	Inhibition of WEE1 impairs HR by indirectly inhibiting BRCA2. Combination therapy resulted in elevated γH2AX, accumulation of DNA damage, and induction of apoptosis.	Mild synergy between WEE1 and PARP inhibition was seen in cell lines harboring *FLT3-ITD*, while *FLT3* wild-type cells were relatively insensitive, independent of *TP53* status. Significantly prolonged survival in a murine model of *FLT3-ITD^+^* AML and reduced colony formation in primary AML samples.	[178]
TRAIL	rTRAIL	Olaparib Veliparib	PARPi upregulate TNFRSF6 and TNFRSF10B expression via potentiation of the Sp1 transcription factor and NF-kB, increasing sensitivity to TRAIL.	Both olaparib and veliparib enhanced the sensitivity of myeloid cell lines to TRAIL in vitro. Though olaparib had no consistent activity alone, it sensitized most primary AML isolates to TRAIL and reduced colony formation.	[161,179]
Antibody drug conjugates	Gemtuzumab ozogamicin	Olaparib	Calicheamicin induces both SSBs and DSBs, invoking PARP activation.	The IC_50_ value for GO was reduced from 24 to 13 ng/mL when combined with olaparib; the CI was 0.86, indicating synergistic cytotoxicity.	[144]

Abbreviations: 5-FU, 5-fluorouracil; CI, cooperativity index; DSB, double strand break; GO, gentuzumab ozogamicin; LSC, leukemia stem cell; PARPi, PARP inhibitor(s); Ref, reference; rTRAIL, recombinant TNF-related apoptosis-inducing ligand; SSB: single strand break; TKI, tyrosine kinase inhibitor.

**Table 3:** Published Clinical Trials of PARP Inhibitors in Hematologic Malignancies.

Trial	Year	Intervention(s)	Phase	Disease(s) ^a^	N	CRR	ORR	OS ^b^	Ref
NCT01399840	2014	Talazoparib	I	AML/MDS CLL/MCL	25 8	0% 0%	0% 0%	N/A	[180]
NCT01139970	2017	Veliparib + Temozolomide	I	AML	48	17%	33%	5.3	[181]
NCT00588991	2017	Veliparib + Topotecan ± Carboplatin	I	AML, MPN, CMML	99	14%	33%	15.3 ^c^	[182]
ISRCTN34386131	2017	Olaparib	I	CLL, MCL, T-PLL	15	0%	0%	4.3	[183]

^a^ All disease groups are relapsed/refractory unless otherwise specified. ^b^ Overall survival reported as median months. ^c^ For patients who responded to therapy. Abbreviations: AML, acute myeloid leukemia; CLL, chronic lymphocytic leukemia; CMML, chronic myelomonocytic leukemia; CRR, complete response rate; MDS, myelodysplastic syndrome; MCL, mantle cell lymphoma; MPN: myeloproliferative neoplasm; N, number of patients; ORR, overall response rate; OS, overall survival; Ref, reference(s).

**Table 4:** Current Clinical Trials of PARP Inhibitors in Myeloid Neoplasms.

Trial	Phase	Intervention(s)	Population(s) ^a^	Status
NCT03289910	II	Topotecan + Carboplatin ± Veliparib	AML, MDS, MPN, CMML	Active (not recruiting)
NCT02878785	I/II	Talazoparib + Decitabine	AML (phase I) AML, untreated (phase II)	Active (not recruiting)
NCT03953898	II	Olaparib	IDH1/2-mutant AML/MDS	Recruiting
NCT03974217	I	Talazoparib	Cohesin-mutant AML/MDS	Recruiting

^a^ All disease groups are relapsed/refractory unless otherwise specified. Abbreviations: AML, acute myeloid leukemia; CLL, chronic lymphocytic leukemia; CMML, chronic myelomonocytic leukemia; MDS, myelodysplastic syndrome; MCL, mantle cell lymphoma; MPN: myeloproliferative neoplasm; T-PLL, T-prolymphocytic leukemia.

**Table 5:** Recent Studies of Therapy-Related Myeloid Neoplasms with PARP Inhibitors.

Authors	N	PARPi	Myeloid Neoplasm	SOT	Karyotype	NGS	SOT Status at Diagnosis	Median OS
Martin et al. [238]	20	Olaparib (94%) Rucaparib (6%)	AML (45%) MDS (55%)	Ovarian	95% complex	DDR pathway mutations in 83%	55% in CR	4.3 months
Kwan et al. [239]	22	Rucaparib	AML 41% MDS 59% *	Ovarian	53% complex; 80% with chrom. 5 or 7 alteration	NR	NR	NR
Morice et al. [19]	178	Olaparib (75%) Niraparib (18%) Rucaparib (6%) Talazoparib (1%) Veliparib (1%)	AML (44%) MDS (56%)	Ovarian (85%) Prostate (7%) Breast (5%) Pancreatic (2%)	NR	NR	Response (85%) Progression (15%)	NR (45% had died on follow-up)

N, number of patients; AML, acute myeloid leukemia; chrom., chromosome; CR, complete remission; DDR, DNA damage response; MDS, myelodysplastic syndrome; SOT, solid organ tumor; NGS, next generation sequencing; OS, overall survival; NR, not reported. * Two patients (9%) presented with MDS and progressed to AML.

## Data Availability

No new data were created or analyzed in this study. Data sharing is not applicable to this article.

## References

[1] Tempero M.A. (2019). NCCN guidelines updates: Pancreatic cancer. J. Natl. Compr. Cancer Netw..

[2] Armstrong D.K., Alvarez R.D., Bakkum-Gamez J.N., Barroilhet L., Behbakht K., Berchuck A., Berek J.S., Chen L.M., Cristea M., DeRosa M. (2019). Ovarian cancer, version 1.2019 featured updates to the nccn guidelines. J. Natl. Compr. Cancer Netw..

[3] Gradishar W.J., Moran M.S., Abraham J., Aft R., Agnese D., Allison K.H., Blair S.L., Burstein H.J., Dang C., Elias A.D. (2021). NCCN guidelines^®^ insights: Breast cancer, version 4.2021: Featured updates to the NCCN guidelines. J. Natl. Compr. Cancer Netw..

[4] Tempero M.A., Malafa M.P., Al-Hawary M., Behrman S.W., Benson A.B., Cardin D.B., Chiorean E.G., Chung V., Czito B., Del Chiaro M. (2021). Pancreatic adenocarcinoma, version 2.2021, NCCN clinical practice guidelines in oncology. J. Natl. Compr. Cancer Netw..

[5] Armstrong D.K., Alvarez R.D., Bakkum-Gamez J.N., Barroilhet L., Behbakht K., Berchuck A., Chen L.-M., Cristea M., DeRosa M., Eisenhauer E.L. (2021). Ovarian cancer, version 2.2020, NCCN clinical practice guidelines in oncology. J. Natl. Compr. Cancer Netw..

[6] Schaeffer E., Srinivas S., Antonarakis E.S., Armstrong A.J., Bekelman J.E., Cheng H., D’Amico A.V., Davis B.J., Desai N., Dorff T. (2021). NCCN guidelines insights: Prostate cancer, version 1.2021: Featured updates to the NCCN guidelines. J. Natl. Compr. Cancer Netw..

[7] Farmer H., McCabe N., Lord C.J., Tutt A.N., Johnson D.A., Richardson T.B., Santarosa M., Dillon K.J., Hickson I., Knights C. (2005). Targeting the DNA repair defect in BRCA mutant cells as a therapeutic strategy. Nature.

[8] Bryant H.E., Schultz N., Thomas H.D., Parker K.M., Flower D., Lopez E., Kyle S., Meuth M., Curtin N.J., Helleday T. (2005). Specific killing of BRCA2-deficient tumours with inhibitors of poly(ADP-ribose) polymerase. Nature.

[9] Li X., Heyer W.D. (2008). Homologous recombination in DNA repair and DNA damage tolerance. Cell Res..

[10] Patel A.G., Sarkaria J.N., Kaufmann S.H. (2011). Nonhomologous end joining drives poly(ADP-ribose) polymerase (PARP) inhibitor lethality in homologous recombination-deficient cells. Proc. Natl. Acad. Sci. USA.

[11] De Lorenzo S.B., Patel A.G., Hurley R.M., Kaufmann S.H. (2013). The elephant and the blind men: Making sense of PARP inhibitors in homologous recombination deficient tumor cells. Front. Oncol..

[12] Kaufman B., Shapira-Frommer R., Schmutzler R.K., Audeh M.W., Friedlander M., Balmaña J., Mitchell G., Fried G., Stemmer S.M., Hubert A. (2015). Olaparib monotherapy in patients with advanced cancer and a germline BRCA1/2 mutation. J. Clin. Oncol..

[13] Robson M., Im S.A., Senkus E., Xu B., Domchek S.M., Masuda N., Delaloge S., Li W., Tung N., Armstrong A. (2017). Olaparib for metastatic breast cancer in patients with a germline BRCA mutation. N. Engl. J. Med..

[14] Mateo J., Porta N., Bianchini D., McGovern U., Elliott T., Jones R., Syndikus I., Ralph C., Jain S., Varughese M. (2020). Olaparib in patients with metastatic castration-resistant prostate cancer with DNA repair gene aberrations (TOPARP-B): A multicentre, open-label, randomised, phase 2 trial. Lancet Oncol..

[15] McCabe N., Turner N.C., Lord C.J., Kluzek K., Bialkowska A., Swift S., Giavara S., O’Connor M.J., Tutt A.N., Zdzienicka M.Z. (2006). Deficiency in the repair of DNA damage by homologous recombination and sensitivity to poly(ADP-ribose) polymerase inhibition. Cancer Res..

[16] Coleman R.L., Oza A.M., Lorusso D., Aghajanian C., Oaknin A., Dean A., Colombo N., Weberpals J.I., Clamp A., Scambia G. (2017). Rucaparib maintenance treatment for recurrent ovarian carcinoma after response to platinum therapy (ARIEL3): A randomised, double-blind, placebo-controlled, phase 3 trial. Lancet.

[17] Mirza M.R., Monk B.J., Herrstedt J., Oza A.M., Mahner S., Redondo A., Fabbro M., Ledermann J.A., Lorusso D., Vergote I. (2016). Niraparib maintenance therapy in platinum-sensitive, recurrent ovarian cancer. N. Engl. J. Med..

[18] Poveda A., Floquet A., Ledermann J.A., Asher R., Penson R.T., Oza A.M., Korach J., Huzarski T., Pignata S., Friedlander M. (2021). Olaparib tablets as maintenance therapy in patients with platinum-sensitive relapsed ovarian cancer and a BRCA1/2 mutation (SOLO2/ENGOT-Ov21): A final analysis of a double-blind, randomised, placebo-controlled, phase 3 trial. Lancet Oncol..

[19] Morice P.M., Leary A., Dolladille C., Chrétien B., Poulain L., González-Martín A., Moore K., O’Reilly E.M., Ray-Coquard I., Alexandre J. (2021). Myelodysplastic syndrome and acute myeloid leukaemia in patients treated with PARP inhibitors: A safety meta-analysis of randomised controlled trials and a retrospective study of the WHO pharmacovigilance database. Lancet Haematol..

[20] Amé J.C., Spenlehauer C., de Murcia G. (2004). The PARP superfamily. Bioessays.

[21] Gagné J.-P., Ethier C., Defoy D., Bourassa S., Langelier M.-F., Riccio A.A., Pascal J.M., Moon K.-M., Foster L.J., Ning Z. (2015). Quantitative site-specific ADP-ribosylation profiling of DNA-dependent PARPs. DNA Repair.

[22] Jungmichel S., Rosenthal F., Altmeyer M., Lukas J., Hottiger M.O., Nielsen M.L. (2013). Proteome-wide identification of poly (ADP-Ribosyl) ation targets in different genotoxic stress responses. Mol. Cell.

[23] Gagne J.-P., Pic E., Isabelle M., Krietsch J., Ethier C., Paquet É., Kelly I., Boutin M., Moon K.-M., Foster L.J. (2012). Quantitative proteomics profiling of the poly (ADP-ribose)-related response to genotoxic stress. Nucleic Acids Res..

[24] Gibson B.A., Zhang Y., Jiang H., Hussey K.M., Shrimp J.H., Lin H., Schwede F., Yu Y., Kraus W.L. (2016). Chemical genetic discovery of PARP targets reveals a role for PARP-1 in transcription elongation. Science.

[25] Haince J.F., McDonald D., Rodrigue A., Déry U., Masson J.Y., Hendzel M.J., Poirier G.G. (2008). PARP1-dependent kinetics of recruitment of MRE11 and NBS1 proteins to multiple DNA damage sites. J. Biol. Chem..

[26] Caron M.C., Sharma A.K., O’Sullivan J., Myler L.R., Ferreira M.T., Rodrigue A., Coulombe Y., Ethier C., Gagné J.P., Langelier M.F. (2019). Poly(ADP-ribose) polymerase-1 antagonizes DNA resection at double-strand breaks. Nat. Commun..

[27] Satoh M.S., Lindahl T. (1992). Role of poly(ADP-ribose) formation in DNA repair. Nature.

[28] Murai J., Huang S.Y., Das B.B., Renaud A., Zhang Y., Doroshow J.H., Ji J., Takeda S., Pommier Y. (2012). Trapping of PARP1 and PARP2 by clinical PARP inhibitors. Cancer Res..

[29] Ray-Coquard I., Pautier P., Pignata S., Pérol D., González-Martín A., Berger R., Fujiwara K., Vergote I., Colombo N., Mäenpää J. (2019). Olaparib plus bevacizumab as first-line maintenance in ovarian cancer. N. Engl. J. Med..

[30] Moore K., Colombo N., Scambia G., Kim B.-G., Oaknin A., Friedlander M., Lisyanskaya A., Floquet A., Leary A., Sonke G.S. (2018). Maintenance olaparib in patients with newly diagnosed advanced ovarian cancer. N. Engl. J. Med..

[31] Pujade-Lauraine E., Ledermann J.A., Selle F., Gebski V., Penson R.T., Oza A.M., Korach J., Huzarski T., Poveda A., Pignata S. (2017). Olaparib tablets as maintenance therapy in patients with platinum-sensitive, relapsed ovarian cancer and a BRCA1/2 mutation (SOLO2/ENGOT-Ov21): A double-blind, randomised, placebo-controlled, phase 3 trial. Lancet Oncol..

[32] Golan T., Hammel P., Reni M., Van Cutsem E., Macarulla T., Hall M.J., Park J.-O., Hochhauser D., Arnold D., Oh D.-Y. (2019). Maintenance olaparib for germline BRCA-mutated metastatic pancreatic cancer. N. Engl. J. Med..

[33] de Bono J., Mateo J., Fizazi K., Saad F., Shore N., Sandhu S., Chi K.N., Sartor O., Agarwal N., Olmos D. (2020). Olaparib for metastatic castration-resistant prostate cancer. N. Engl. J. Med..

[34] Hussain M., Mateo J., Fizazi K., Saad F., Shore N., Sandhu S., Chi K.N., Sartor O., Agarwal N., Olmos D. (2020). Survival with olaparib in metastatic castration-resistant prostate cancer. N. Engl. J. Med..

[35] Ley T.J., Miller C., Ding L., Raphael B.J., Mungall A.J., Robertson A., Hoadley K., Triche T.J., Laird P.W., Baty J.D. (2013). Genomic and epigenomic landscapes of adult de novo acute myeloid leukemia. N. Engl. J. Med..

[36] Karp J.E., Thomas B.M., Greer J.M., Sorge C., Gore S.D., Pratz K.W., Smith B.D., Flatten K.S., Peterson K., Schneider P. (2012). Phase I and pharmacologic trial of cytosine arabinoside with the selective checkpoint 1 inhibitor Sch 900776 in refractory acute leukemias. Clin. Cancer Res..

[37] Suarez F., Mahlaoui N., Canioni D., Andriamanga C., Dubois d’Enghien C., Brousse N., Jais J.-P., Fischer A., Hermine O., Stoppa-Lyonnet D. (2015). Incidence, presentation, and prognosis of malignancies in ataxia-telangiectasia: A report from the French national registry of primary immune deficiencies. J. Clin. Oncol..

[38] Esposito M.T., Zhao L., Fung T.K., Rane J.K., Wilson A., Martin N., Gil J., Leung A.Y., Ashworth A., So C.W. (2015). Synthetic lethal targeting of oncogenic transcription factors in acute leukemia by PARP inhibitors. Nat. Med..

[39] Alcalay M., Meani N., Gelmetti V., Fantozzi A., Fagioli M., Orleth A., Riganelli D., Sebastiani C., Cappelli E., Casciari C. (2003). Acute myeloid leukemia fusion proteins deregulate genes involved in stem cell maintenance and DNA repair. J. Clin. Investig..

[40] Faraoni I., Compagnone M., Lavorgna S., Angelini D.F., Cencioni M.T., Piras E., Panetta P., Ottone T., Dolci S., Venditti A. (2015). BRCA1, PARP1 and γH2AX in acute myeloid leukemia: Role as biomarkers of response to the PARP inhibitor olaparib. Biochim. Biophys. Acta.

[41] Zhao L., So C.W. (2016). PARP-inhibitor-induced synthetic lethality for acute myeloid leukemia treatment. Exp. Hematol..

[42] Krejci O., Wunderlich M., Geiger H., Chou F.S., Schleimer D., Jansen M., Andreassen P.R., Mulloy J.C. (2008). p53 signaling in response to increased DNA damage sensitizes AML1-ETO cells to stress-induced death. Blood.

[43] Fontana M.C., Marconi G., Feenstra J.D.M., Fonzi E., Papayannidis C., Ghelli Luserna di Rorá A., Padella A., Solli V., Franchini E., Ottaviani E. (2018). Chromothripsis in acute myeloid leukemia: Biological features and impact on survival. Leukemia.

[44] Gaymes T.J., Mufti G.J., Rassool F.V. (2002). Myeloid leukemias have increased activity of the nonhomologous end-joining pathway and concomitant DNA misrepair that is dependent on the Ku70/86 heterodimer. Cancer Res..

[45] Gibson B.A., Kraus W.L. (2012). New insights into the molecular and cellular functions of poly(ADP-ribose) and PARPs. Nat. Rev. Mol. Cell Biol..

[46] Krietsch J., Rouleau M., Pic É., Ethier C., Dawson T.M., Dawson V.L., Masson J.Y., Poirier G.G., Gagné J.P. (2013). Reprogramming cellular events by poly(ADP-ribose)-binding proteins. Mol. Aspects Med..

[47] Messner S., Hottiger M.O. (2011). Histone ADP-ribosylation in DNA repair, replication and transcription. Trends Cell Biol..

[48] Realini C.A., Althaus F.R. (1992). Histone shuttling by poly(ADP-ribosylation). J. Biol. Chem..

[49] Kraus W.L., Hottiger M.O. (2013). PARP-1 and gene regulation: Progress and puzzles. Mol. Aspects Med..

[50] Quénet D., El Ramy R., Schreiber V., Dantzer F. (2009). The role of poly(ADP-ribosyl)ation in epigenetic events. Int. J. Biochem. Cell Biol..

[51] Wang T., Simbulan-Rosenthal C.M., Smulson M.E., Chock P.B., Yang D.C. (2008). Polyubiquitylation of PARP-1 through ubiquitin K48 is modulated by activated DNA, NAD+, and dipeptides. J. Cell. Biochem..

[52] Wang Z., Michaud G.A., Cheng Z., Zhang Y., Hinds T.R., Fan E., Cong F., Xu W. (2012). Recognition of the iso-ADP-ribose moiety in poly(ADP-ribose) by WWE domains suggests a general mechanism for poly(ADP-ribosyl)ation-dependent ubiquitination. Genes Dev..

[53] Aravind L. (2001). The WWE domain: A common interaction module in protein ubiquitination and ADP ribosylation. Trends Biochem. Sci..

[54] Luo X., Kraus W.L. (2012). On PAR with PARP: Cellular stress signaling through poly(ADP-ribose) and PARP-1. Genes Dev..

[55] Bai P., Cantó C. (2012). The role of PARP-1 and PARP-2 enzymes in metabolic regulation and disease. Cell Metab..

[56] Dantzer F., Schreiber V., Niedergang C., Trucco C., Flatter E., De La Rubia G., Oliver J., Rolli V., Ménissier-de Murcia J., de Murcia G. (1999). Involvement of poly(ADP-ribose) polymerase in base excision repair. Biochimie.

[57] De Murcia J.M., Niedergang C., Trucco C., Ricoul M., Dutrillaux B., Mark M., Oliver F.J., Masson M., Dierich A., LeMeur M. (1997). Requirement of poly(ADP-ribose) polymerase in recovery from DNA damage in mice and in cells. Proc. Natl. Acad. Sci. USA.

[58] Masson M., Niedergang C., Schreiber V., Muller S., Menissier-de Murcia J., de Murcia G. (1998). XRCC1 is specifically associated with poly(ADP-ribose) polymerase and negatively regulates its activity following DNA damage. Mol. Cell. Biol..

[59] Trucco C., Oliver F.J., de Murcia G., Ménissier-de Murcia J. (1998). DNA repair defect in poly(ADP-ribose) polymerase-deficient cell lines. Nucleic Acids Res..

[60] Schultz N., Lopez E., Saleh-Gohari N., Helleday T. (2003). Poly(ADP-ribose) polymerase (PARP-1) has a controlling role in homologous recombination. Nucleic Acids Res..

[61] Langelier M.F., Pascal J.M. (2013). PARP-1 mechanism for coupling DNA damage detection to poly(ADP-ribose) synthesis. Curr. Opin. Struct. Biol..

[62] Langelier M.F., Planck J.L., Roy S., Pascal J.M. (2012). Structural basis for DNA damage-dependent poly(ADP-ribosyl)ation by human PARP-1. Science.

[63] Hassa P.O., Hottiger M.O. (2008). The diverse biological roles of mammalian PARPS, a small but powerful family of poly-ADP-ribose polymerases. Front. Biosci..

[64] Gradwohl G., Ménissier de Murcia J.M., Molinete M., Simonin F., Koken M., Hoeijmakers J.H., de Murcia G. (1990). The second zinc-finger domain of poly(ADP-ribose) polymerase determines specificity for single-stranded breaks in DNA. Proc. Natl. Acad. Sci. USA.

[65] Kulczyk A.W., Yang J.C., Neuhaus D. (2004). Solution structure and DNA binding of the zinc-finger domain from DNA ligase IIIalpha. J. Mol. Biol..

[66] Altmeyer M., Messner S., Hassa P.O., Fey M., Hottiger M.O. (2009). Molecular mechanism of poly(ADP-ribosyl)ation by PARP1 and identification of lysine residues as ADP-ribose acceptor sites. Nucleic Acids Res..

[67] Langelier M.F., Planck J.L., Roy S., Pascal J.M. (2011). Crystal structures of poly(ADP-ribose) polymerase-1 (PARP-1) zinc fingers bound to DNA: Structural and functional insights into DNA-dependent PARP-1 activity. J. Biol. Chem..

[68] Pion E., Bombarda E., Stiegler P., Ullmann G.M., Mély Y., de Murcia G., Gérard D. (2003). Poly(ADP-ribose) polymerase-1 dimerizes at a 5’ recessed DNA end in vitro: A fluorescence study. Biochemistry.

[69] Pion E., Ullmann G.M., Amé J.C., Gérard D., de Murcia G., Bombarda E. (2005). DNA-induced dimerization of poly(ADP-ribose) polymerase-1 triggers its activation. Biochemistry.

[70] Tao Z., Gao P., Liu H.W. (2009). Identification of the ADP-ribosylation sites in the PARP-1 automodification domain: Analysis and implications. J. Am. Chem. Soc..

[71] Mendoza-Alvarez H., Alvarez-Gonzalez R. (1993). Poly(ADP-ribose) polymerase is a catalytic dimer and the automodification reaction is intermolecular. J. Biol. Chem..

[72] Schreiber V., Amé J.C., Dollé P., Schultz I., Rinaldi B., Fraulob V., Ménissier-de Murcia J., de Murcia G. (2002). Poly(ADP-ribose) polymerase-2 (PARP-2) is required for efficient base excision DNA repair in association with PARP-1 and XRCC1. J. Biol. Chem..

[73] Prokhorova E., Zobel F., Smith R., Zentout S., Gibbs-Seymour I., Schutzenhofer K., Peters A., Groslambert J., Zorzini V., Agnew T. (2021). Serine-linked PARP1 auto-modification controls PARP inhibitor response. Nat. Commun..

[74] Suskiewicz M.J., Zobel F., Ogden T.E.H., Fontana P., Ariza A., Yang J.C., Zhu K., Bracken L., Hawthorne W.J., Ahel D. (2020). HPF1 completes the PARP active site for DNA damage-induced ADP-ribosylation. Nature.

[75] Hendriks I.A., Buch-Larsen S.C., Prokhorova E., Elsborg J.D., Rebak A., Zhu K., Ahel D., Lukas C., Ahel I., Nielsen M.L. (2021). The regulatory landscape of the human HPF1- and ARH3-dependent ADP-ribosylome. Nat. Commun..

[76] Li M., Yu X. (2013). Function of BRCA1 in the DNA damage response is mediated by ADP-ribosylation. Cancer Cell.

[77] Min W., Bruhn C., Grigaravicius P., Zhou Z.W., Li F., Krüger A., Siddeek B., Greulich K.O., Popp O., Meisezahl C. (2013). Poly(ADP-ribose) binding to Chk1 at stalled replication forks is required for S-phase checkpoint activation. Nat. Commun..

[78] Hu Y., Petit S.A., Ficarro S.B., Toomire K.J., Xie A., Lim E., Cao S.A., Park E., Eck M.J., Scully R. (2014). PARP1-driven poly-ADP-ribosylation regulates BRCA1 function in homologous recombination-mediated DNA repair. Cancer Discov..

[79] Otto H., Reche P.A., Bazan F., Dittmar K., Haag F., Koch-Nolte F. (2005). In silico characterization of the family of PARP-like poly(ADP-ribosyl)transferases (pARTs). BMC Genom..

[80] Bell C.E., Eisenberg D. (1996). Crystal structure of diphtheria toxin bound to nicotinamide adenine dinucleotide. Biochemistry.

[81] Kleine H., Poreba E., Lesniewicz K., Hassa P.O., Hottiger M.O., Litchfield D.W., Shilton B.H., Lüscher B. (2008). Substrate-assisted catalysis by PARP10 limits its activity to mono-ADP-ribosylation. Mol. Cell.

[82] Rouleau M., Patel A., Hendzel M.J., Kaufmann S.H., Poirier G.G. (2010). PARP inhibition: PARP1 and beyond. Nat. Rev. Cancer.

[83] Juarez-Salinas H., Sims J.L., Jacobson M.K. (1979). Poly(ADP-ribose) levels in carcinogen-treated cells. Nature.

[84] Wang M., Wu W., Wu W., Rosidi B., Zhang L., Wang H., Iliakis G. (2006). PARP-1 and Ku compete for repair of DNA double strand breaks by distinct NHEJ pathways. Nucleic Acids Res..

[85] Robert I., Dantzer F., Reina-San-Martin B. (2009). Parp1 facilitates alternative NHEJ, whereas Parp2 suppresses IgH/c-myc translocations during immunoglobulin class switch recombination. J. Exp. Med..

[86] Soni A., Siemann M., Grabos M., Murmann T., Pantelias G.E., Iliakis G. (2014). Requirement for Parp-1 and DNA ligases 1 or 3 but not of Xrcc1 in chromosomal translocation formation by backup end joining. Nucleic Acids Res..

[87] Bryant H.E., Petermann E., Schultz N., Jemth A.S., Loseva O., Issaeva N., Johansson F., Fernandez S., McGlynn P., Helleday T. (2009). PARP is activated at stalled forks to mediate Mre11-dependent replication restart and recombination. EMBO J..

[88] Hottiger M.O., Hassa P.O., Lüscher B., Schüler H., Koch-Nolte F. (2010). Toward a unified nomenclature for mammalian ADP-ribosyltransferases. Trends Biochem. Sci..

[89] Amé J.C., Rolli V., Schreiber V., Niedergang C., Apiou F., Decker P., Muller S., Höger T., Ménissier-de Murcia J., de Murcia G. (1999). PARP-2, A novel mammalian DNA damage-dependent poly(ADP-ribose) polymerase. J. Biol. Chem..

[90] Rulten S.L., Fisher A.E., Robert I., Zuma M.C., Rouleau M., Ju L., Poirier G., Reina-San-Martin B., Caldecott K.W. (2011). PARP-3 and APLF function together to accelerate nonhomologous end-joining. Mol. Cell.

[91] Boehler C., Gauthier L.R., Mortusewicz O., Biard D.S., Saliou J.M., Bresson A., Sanglier-Cianferani S., Smith S., Schreiber V., Boussin F. (2011). Poly(ADP-ribose) polymerase 3 (PARP3), a newcomer in cellular response to DNA damage and mitotic progression. Proc. Natl. Acad. Sci. USA.

[92] Wahlberg E., Karlberg T., Kouznetsova E., Markova N., Macchiarulo A., Thorsell A.G., Pol E., Frostell Å., Ekblad T., Öncü D. (2012). Family-wide chemical profiling and structural analysis of PARP and tankyrase inhibitors. Nat. Biotechnol..

[93] Thorsell A.G., Ekblad T., Karlberg T., Löw M., Pinto A.F., Trésaugues L., Moche M., Cohen M.S., Schüler H. (2017). Structural basis for potency and promiscuity in poly(ADP-ribose) polymerase (PARP) and tankyrase inhibitors. J. Med. Chem..

[94] Haince J.F., Kozlov S., Dawson V.L., Dawson T.M., Hendzel M.J., Lavin M.F., Poirier G.G. (2007). Ataxia telangiectasia mutated (ATM) signaling network is modulated by a novel poly(ADP-ribose)-dependent pathway in the early response to DNA-damaging agents. J. Biol. Chem..

[95] Zaremba T., Curtin N.J. (2007). PARP inhibitor development for systemic cancer targeting. Anticancer Agents Med. Chem..

[96] Ratnam K., Low J.A. (2007). Current development of clinical inhibitors of poly(ADP-ribose) polymerase in oncology. Clin. Cancer Res..

[97] Scott C.L., Swisher E.M., Kaufmann S.H. (2015). Poly (ADP-ribose) polymerase inhibitors: Recent advances and future development. J. Clin. Oncol..

[98] Schreiber V., Dantzer F., Ame J.C., de Murcia G. (2006). Poly(ADP-ribose): Novel functions for an old molecule. Nat. Rev. Mol. Cell Biol..

[99] Saleh-Gohari N., Bryant H.E., Schultz N., Parker K.M., Cassel T.N., Helleday T. (2005). Spontaneous homologous recombination is induced by collapsed replication forks that are caused by endogenous DNA single-strand breaks. Mol. Cell. Biol..

[100] Fong P.C., Boss D.S., Yap T.A., Tutt A., Wu P., Mergui-Roelvink M., Mortimer P., Swaisland H., Lau A., O’Connor M.J. (2009). Inhibition of poly(ADP-ribose) polymerase in tumors from BRCA mutation carriers. N. Engl. J. Med..

[101] Gottipati P., Vischioni B., Schultz N., Solomons J., Bryant H.E., Djureinovic T., Issaeva N., Sleeth K., Sharma R.A., Helleday T. (2010). Poly(ADP-ribose) polymerase is hyperactivated in homologous recombination-defective cells. Cancer Res..

[102] Wang Z.Q., Auer B., Stingl L., Berghammer H., Haidacher D., Schweiger M., Wagner E.F. (1995). Mice lacking ADPRT and poly(ADP-ribosyl)ation develop normally but are susceptible to skin disease. Genes Dev..

[103] Liu X., Han E.K., Anderson M., Shi Y., Semizarov D., Wang G., McGonigal T., Roberts L., Lasko L., Palma J. (2009). Acquired resistance to combination treatment with temozolomide and ABT-888 is mediated by both base excision repair and homologous recombination DNA repair pathways. Mol. Cancer Res..

[104] Patel A.G., Flatten K.S., Schneider P.A., Dai N.T., McDonald J.S., Poirier G.G., Kaufmann S.H. (2012). Enhanced killing of cancer cells by poly(ADP-ribose) polymerase inhibitors and topoisomerase inhibitors reflects poisoning of both enzymes. J. Biol. Chem..

[105] Gagné J.P., Isabelle M., Lo K.S., Bourassa S., Hendzel M.J., Dawson V.L., Dawson T.M., Poirier G.G. (2008). Proteome-wide identification of poly(ADP-ribose) binding proteins and poly(ADP-ribose)-associated protein complexes. Nucleic Acids Res..

[106] Küpper J.H., de Murcia G., Bürkle A. (1990). Inhibition of poly(ADP-ribosyl)ation by overexpressing the poly(ADP-ribose) polymerase DNA-binding domain in mammalian cells. J. Biol. Chem..

[107] Molinete M., Vermeulen W., Bürkle A., Ménissier-de Murcia J., Küpper J.H., Hoeijmakers J.H., de Murcia G. (1993). Overproduction of the poly(ADP-ribose) polymerase DNA-binding domain blocks alkylation-induced DNA repair synthesis in mammalian cells. EMBO J..

[108] Bonner W.M., Redon C.E., Dickey J.S., Nakamura A.J., Sedelnikova O.A., Solier S., Pommier Y. (2008). GammaH2AX and cancer. Nat. Rev. Cancer.

[109] Celeste A., Fernandez-Capetillo O., Kruhlak M.J., Pilch D.R., Staudt D.W., Lee A., Bonner R.F., Bonner W.M., Nussenzweig A. (2003). Histone H2AX phosphorylation is dispensable for the initial recognition of DNA breaks. Nat. Cell Biol..

[110] Chang H.H.Y., Pannunzio N.R., Adachi N., Lieber M.R. (2017). Non-homologous DNA end joining and alternative pathways to double-strand break repair. Nat. Rev. Mol. Cell Biol..

[111] Paddock M.N., Bauman A.T., Higdon R., Kolker E., Takeda S., Scharenberg A.M. (2011). Competition between PARP-1 and Ku70 control the decision between high-fidelity and mutagenic DNA repair. DNA Repair.

[112] Hochegger H., Dejsuphong D., Fukushima T., Morrison C., Sonoda E., Schreiber V., Zhao G.Y., Saberi A., Masutani M., Adachi N. (2006). Parp-1 protects homologous recombination from interference by Ku and Ligase IV in vertebrate cells. EMBO J..

[113] Ceccaldi R., Liu J.C., Amunugama R., Hajdu I., Primack B., Petalcorin M.I., O’Connor K.W., Konstantinopoulos P.A., Elledge S.J., Boulton S.J. (2015). Homologous-recombination-deficient tumours are dependent on Polθ-mediated repair. Nature.

[114] Murai J., Yang K., Dejsuphong D., Hirota K., Takeda S., D’Andrea A.D. (2011). The USP1/UAF1 complex promotes double-strand break repair through homologous recombination. Mol. Cell. Biol..

[115] Audebert M., Salles B., Calsou P. (2004). Involvement of poly(ADP-ribose) polymerase-1 and XRCC1/DNA ligase III in an alternative route for DNA double-strand breaks rejoining. J. Biol. Chem..

[116] Mateos-Gomez P.A., Gong F., Nair N., Miller K.M., Lazzerini-Denchi E., Sfeir A. (2015). Mammalian polymerase θ promotes alternative NHEJ and suppresses recombination. Nature.

[117] Zhou J., Gelot C., Pantelidou C., Li A., Yücel H., Davis R.E., Farkkila A., Kochupurakkal B., Syed A., Shapiro G.I. (2021). A first-in-class polymerase theta inhibitor selectively targets homologous-recombination-deficient tumors. Nat. Cancer.

[118] Ray Chaudhuri A., Callen E., Ding X., Gogola E., Duarte A.A., Lee J.E., Wong N., Lafarga V., Calvo J.A., Panzarino N.J. (2016). Replication fork stability confers chemoresistance in BRCA-deficient cells. Nature.

[119] Schlacher K., Christ N., Siaud N., Egashira A., Wu H., Jasin M. (2011). Double-strand break repair-independent role for BRCA2 in blocking stalled replication fork degradation by MRE11. Cell.

[120] Ying S., Hamdy F.C., Helleday T. (2012). Mre11-dependent degradation of stalled DNA replication forks is prevented by BRCA2 and PARP1. Cancer Res..

[121] Yang Y.G., Cortes U., Patnaik S., Jasin M., Wang Z.Q. (2004). Ablation of PARP-1 does not interfere with the repair of DNA double-strand breaks, but compromises the reactivation of stalled replication forks. Oncogene.

[122] Malanga M., Althaus F.R. (2004). Poly(ADP-ribose) reactivates stalled DNA topoisomerase I and Induces DNA strand break resealing. J. Biol. Chem..

[123] Ruf A., Mennissier de Murcia J., de Murcia G., Schulz G.E. (1996). Structure of the catalytic fragment of poly(AD-ribose) polymerase from chicken. Proc. Natl. Acad. Sci. USA.

[124] Ruf A., de Murcia G., Schulz G.E. (1998). Inhibitor and NAD+ binding to poly(ADP-ribose) polymerase as derived from crystal structures and homology modeling. Biochemistry.

[125] Lawlor D., Martin P., Busschots S., Thery J., O’Leary J.J., Hennessy B.T., Stordal B. (2014). PARP Inhibitors as P-glyoprotein Substrates. J. Pharm. Sci..

[126] Henneman L., van Miltenburg M.H., Michalak E.M., Braumuller T.M., Jaspers J.E., Drenth A.P., de Korte-Grimmerink R., Gogola E., Szuhai K., Schlicker A. (2015). Selective resistance to the PARP inhibitor olaparib in a mouse model for BRCA1-deficient metaplastic breast cancer. Proc. Natl. Acad. Sci. USA.

[127] Rudolph J., Roberts G., Luger K. (2021). Histone parylation factor 1 contributes to the inhibition of PARP1 by cancer drugs. Nat. Commun..

[128] Menear K.A., Adcock C., Boulter R., Cockcroft X.L., Copsey L., Cranston A., Dillon K.J., Drzewiecki J., Garman S., Gomez S. (2008). 4-[3-(4-cyclopropanecarbonylpiperazine-1-carbonyl)-4-fluorobenzyl]-2H-phthalazin-1-one: A novel bioavailable inhibitor of poly(ADP-ribose) polymerase-1. J. Med. Chem..

[129] Wang B., Chu D., Feng Y., Shen Y., Aoyagi-Scharber M., Post L.E. (2016). Discovery and characterization of (8*S*,9*R*)-5-Fluoro-8-(4-fluorophenyl)-9-(1-methyl-1*H*-1,2,4-triazol-5-yl)-2,7,8,9-tetrahydro-3*H*-pyrido [4,3,2-de]phthalazin-3-one (BMN 673, Talazoparib), a novel, highly potent, and orally efficacious poly(ADP-ribose) polymerase-1/2 inhibitor, as an anticancer agent. J. Med. Chem..

[130] Murai J., Huang S.Y., Renaud A., Zhang Y., Ji J., Takeda S., Morris J., Teicher B., Doroshow J.H., Pommier Y. (2014). Stereospecific PARP trapping by BMN 673 and comparison with olaparib and rucaparib. Mol. Cancer Ther..

[131] Thomas H.D., Calabrese C.R., Batey M.A., Canan S., Hostomsky Z., Kyle S., Maegley K.A., Newell D.R., Skalitzky D., Wang L.Z. (2007). Preclinical selection of a novel poly(ADP-ribose) polymerase inhibitor for clinical trial. Mol. Cancer Ther..

[132] Xie Z., Zhou Y., Zhao W., Jiao H., Chen Y., Yang Y., Li Z. (2015). Identification of novel PARP-1 inhibitors: Drug design, synthesis and biological evaluation. Bioorgan. Med. Chem. Lett..

[133] Jones P., Altamura S., Boueres J., Ferrigno F., Fonsi M., Giomini C., Lamartina S., Monteagudo E., Ontoria J.M., Orsale M.V. (2009). Discovery of 2-{4-[(3*S*)-piperidin-3-yl]phenyl}-2*H*-indazole-7-carboxamide (MK-4827): A novel oral poly(ADP-ribose)polymerase (PARP) inhibitor efficacious in BRCA-1 and -2 mutant tumors. J. Med. Chem..

[134] Jones P., Wilcoxen K., Rowley M., Toniatti C. (2015). Niraparib: A poly(ADP-ribose) polymerase (PARP) inhibitor for the treatment of tumors with defective homologous recombination. J. Med. Chem..

[135] Donawho C.K., Luo Y., Luo Y., Penning T.D., Bauch J.L., Bouska J.J., Bontcheva-Diaz V.D., Cox B.F., DeWeese T.L., Dillehay L.E. (2007). ABT-888, an orally active poly(ADP-ribose) polymerase inhibitor that potentiates DNA-damaging agents in preclinical tumor models. Clin. Cancer Res..

[136] Wang H., Ren B., Liu Y., Jiang B., Guo Y., Wei M., Luo L., Kuang X., Qiu M., Lv L. (2020). Discovery of Pamiparib (BGB-290), a Potent and Selective Poly (ADP-ribose) Polymerase (PARP) Inhibitor in Clinical Development. J. Med. Chem..

[137] Xiong Y., Guo Y., Liu Y., Wang H., Gong W., Liu Y., Wang X., Gao Y., Yu F., Su D. (2020). Pamiparib is a potent and selective PARP inhibitor with unique potential for the treatment of brain tumor. Neoplasia.

[138] Markham A. (2021). Pamiparib: First approval. Drugs.

[139] Mateo J., Carreira S., Sandhu S., Miranda S., Mossop H., Perez-Lopez R., Nava Rodrigues D., Robinson D., Omlin A., Tunariu N. (2015). DNA-repair defects and olaparib in metastatic prostate cancer. N. Engl. J. Med..

[140] Esposito M.T., So C.W. (2014). DNA damage accumulation and repair defects in acute myeloid leukemia: Implications for pathogenesis, disease progression, and chemotherapy resistance. Chromosoma.

[141] Faraoni I., Giansanti M., Voso M.T., Lo-Coco F., Graziani G. (2019). Targeting ADP-ribosylation by PARP inhibitors in acute myeloid leukaemia and related disorders. Biochem. Pharmacol..

[142] Santos M.A., Faryabi R.B., Ergen A.V., Day A.M., Malhowski A., Canela A., Onozawa M., Lee J.E., Callen E., Gutierrez-Martinez P. (2014). DNA-damage-induced differentiation of leukaemic cells as an anti-cancer barrier. Nature.

[143] Gaymes T.J., Shall S., MacPherson L.J., Twine N.A., Lea N.C., Farzaneh F., Mufti G.J. (2009). Inhibitors of poly ADP-ribose polymerase (PARP) induce apoptosis of myeloid leukemic cells: Potential for therapy of myeloid leukemia and myelodysplastic syndromes. Haematologica.

[144] Yamauchi T., Uzui K., Nishi R., Shigemi H., Ueda T. (2014). Gemtuzumab ozogamicin and olaparib exert synergistic cytotoxicity in CD33-positive HL-60 myeloid leukemia cells. Anticancer Res..

[145] Wang L., Cai W., Zhang W., Chen X., Dong W., Tang D., Zhang Y., Ji C., Zhang M. (2015). Inhibition of poly(ADP-ribose) polymerase 1 protects against acute myeloid leukemia by suppressing the myeloproliferative leukemia virus oncogene. Oncotarget.

[146] Nieborowska-Skorska M., Sullivan K., Dasgupta Y., Podszywalow-Bartnicka P., Hoser G., Maifrede S., Martinez E., Di Marcantonio D., Bolton-Gillespie E., Cramer-Morales K. (2017). Gene expression and mutation-guided synthetic lethality eradicates proliferating and quiescent leukemia cells. J. Clin. Investig..

[147] Podszywalow-Bartnicka P., Wolczyk M., Kusio-Kobialka M., Wolanin K., Skowronek K., Nieborowska-Skorska M., Dasgupta Y., Skorski T., Piwocka K. (2014). Downregulation of BRCA1 protein in BCR-ABL1 leukemia cells depends on stress-triggered TIAR-mediated suppression of translation. Cell Cycle.

[148] Forster V.J., Nahari M.H., Martinez-Soria N., Bradburn A.K., Ptasinska A., Assi S.A., Fordham S.E., McNeil H., Bonifer C., Heidenreich O. (2016). The leukemia-associated RUNX1/ETO oncoprotein confers a mutator phenotype. Leukemia.

[149] Sulkowski P.L., Corso C.D., Robinson N.D., Scanlon S.E., Purshouse K.R., Bai H., Liu Y., Sundaram R.K., Hegan D.C., Fons N.R. (2017). 2-Hydroxyglutarate produced by neomorphic IDH mutations suppresses homologous recombination and induces PARP inhibitor sensitivity. Sci. Transl. Med..

[150] Molenaar R.J., Radivoyevitch T., Nagata Y., Khurshed M., Przychodzen B., Makishima H., Xu M., Bleeker F.E., Wilmink J.W., Carraway H.E. (2018). IDH1/2 mutations sensitize acute myeloid leukemia to PARP inhibition and this is reversed by IDH1/2-mutant inhibitors. Clin. Cancer Res..

[151] Sule A., Van Doorn J., Sundaram R.K., Ganesa S., Vasquez J.C., Bindra R.S. (2021). Targeting IDH1/2 mutant cancers with combinations of ATR and PARP inhibitors. NAR Cancer.

[152] Casorelli I., Tenedini E., Tagliafico E., Blasi M.F., Giuliani A., Crescenzi M., Pelosi E., Testa U., Peschle C., Mele L. (2006). Identification of a molecular signature for leukemic promyelocytes and their normal counterparts: Focus on DNA repair genes. Leukemia.

[153] Tothova Z., Valton A.L., Gorelov R.A., Vallurupalli M., Krill-Burger J.M., Holmes A., Landers C.C., Haydu J.E., Malolepsza E., Hartigan C. (2021). Cohesin mutations alter DNA damage repair and chromatin structure and create therapeutic vulnerabilities in MDS/AML. JCI Insight.

[154] Lang F., Liu Y., Chou F.J., Yang C. (2021). Genotoxic therapy and resistance mechanism in gliomas. Pharmacol. Ther..

[155] Berti M., Ray Chaudhuri A., Thangavel S., Gomathinayagam S., Kenig S., Vujanovic M., Odreman F., Glatter T., Graziano S., Mendoza-Maldonado R. (2013). Human RECQ1 promotes restart of replication forks reversed by DNA topoisomerase I inhibition. Nat. Struct. Mol. Biol..

[156] Muvarak N.E., Chowdhury K., Xia L., Robert C., Choi E.Y., Cai Y., Bellani M., Zou Y., Singh Z.N., Duong V.H. (2016). Enhancing the cytotoxic effects of PARP inhibitors with DNA demethylating agents—A potential therapy for cancer. Cancer Cell.

[157] Kogan A.A., Mclaughlin L.J., Topper M., Muvarak N., Stojanovic L., Creed T.M., Bentzen S., Civin C.I., Baer M.R., Kingsbury T.J. (2017). DNA demethylating agents generate a brcaness effect in multiple sporadic tumor types: Prediction for sensitivity to PARP inhibitors in AML. Blood.

[158] Maifrede S., Nieborowska-Skorska M., Sullivan-Reed K., Dasgupta Y., Podszywalow-Bartnicka P., Le B.V., Solecka M., Lian Z., Belyaeva E.A., Nersesyan A. (2018). Tyrosine kinase inhibitor-induced defects in DNA repair sensitize FLT3(ITD)-positive leukemia cells to PARP1 inhibitors. Blood.

[159] Podszywalow-Bartnicka P., Maifrede S., Le B.V., Nieborowska-Skorska M., Piwocka K., Skorski T. (2019). PARP1 inhibitor eliminated imatinib-refractory chronic myeloid leukemia cells in bone marrow microenvironment conditions. Leuk. Lymphoma.

[160] Nieborowska-Skorska M., Maifrede S., Dasgupta Y., Sullivan K., Flis S., Le B.V., Solecka M., Belyaeva E.A., Kubovcakova L., Nawrocki M. (2017). Ruxolitinib-induced defects in DNA repair cause sensitivity to PARP inhibitors in myeloproliferative neoplasms. Blood.

[161] Meng X.W., Koh B.D., Zhang J.S., Flatten K.S., Schneider P.A., Billadeau D.D., Hess A.D., Smith B.D., Karp J.E., Kaufmann S.H. (2014). Poly(ADP-ribose) polymerase inhibitors sensitize cancer cells to death receptor-mediated apoptosis by enhancing death receptor expression. J. Biol. Chem..

[162] Seedhouse C.H., Hunter H.M., Lloyd-Lewis B., Massip A.M., Pallis M., Carter G.I., Grundy M., Shang S., Russell N.H. (2006). DNA repair contributes to the drug-resistant phenotype of primary acute myeloid leukaemia cells with FLT3 internal tandem duplications and is reversed by the FLT3 inhibitor PKC412. Leukemia.

[163] Bamezai S., He J., Sahin D., Mohr F., Ciccarone F., Vegi N.M., Pulikkottil Jose A., Mulaw M.A., Caiafa P., Döhner K. (2016). The PARP inhibitor olaparib antagonizes leukemic growth induced by TET1 overexpression in AML1-ETO positive acute myeloid leukemia. Blood.

[164] Giansanti M., De Gabrieli A., Prete S.P., Ottone T., Divona M.D., Karimi T., Ciccarone F., Voso M.T., Graziani G., Faraoni I. (2021). Poly(ADP-ribose) polymerase inhibitors for arsenic trioxide-resistant acute promyelocytic leukemia: Synergistic in vitro antitumor effects with hypomethylating agents or high-dose vitamin C. J. Pharmacol. Exp. Ther..

[165] Maifrede S., Martinez E., Nieborowska-Skorska M., Di Marcantonio D., Hulse M., Le B.V., Zhao H., Piwocka K., Tempera I., Sykes S.M. (2017). MLL-AF9 leukemias are sensitive to PARP1 inhibitors combined with cytotoxic drugs. Blood Adv..

[166] Zhao L., So C.W.E. (2017). PARPi potentiates with current conventional therapy in MLL leukemia. Cell Cycle.

[167] Piao J., Takai S., Kamiya T., Inukai T., Sugita K., Ohyashiki K., Delia D., Masutani M., Mizutani S., Takagi M. (2017). Poly (ADP-ribose) polymerase inhibitors selectively induce cytotoxicity in TCF3-HLF-positive leukemic cells. Cancer Lett..

[168] Pratz K.W., Koh B.D., Patel A.G., Flatten K.S., Poh W., Herman J.G., Dilley R., Harrell M.I., Smith B.D., Karp J.E. (2016). Poly (ADP-ribose) polymerase inhibitor hypersensitivity in aggressive myeloproliferative neoplasms. Clin. Cancer Res..

[169] Patel P.R., Senyuk V., Rodriguez N.S., Oh A.L., Bonetti E., Mahmud D., Barosi G., Mahmud N., Rondelli D. (2019). Synergistic cytotoxic effect of busulfan and the PARP inhibitor veliparib in myeloproliferative neoplasms. Biol. Blood Marrow Transplant..

[170] Murai J., Zhang Y., Morris J., Ji J., Takeda S., Doroshow J.H., Pommier Y. (2014). Rationale for poly(ADP-ribose) polymerase (PARP) inhibitors in combination therapy with camptothecins or temozolomide based on PARP trapping versus catalytic inhibition. J. Pharmacol. Exp. Ther..

[171] Falzacappa M.V., Ronchini C., Faretta M., Iacobucci I., Di Rorà A.G., Martinelli G., Meyer L.H., Debatin K.M., Orecchioni S., Bertolini F. (2015). The combination of the PARP inhibitor rucaparib and 5FU is an effective strategy for treating acute leukemias. Mol. Cancer Ther..

[172] Bowman K.J., White A., Golding B.T., Griffin R.J., Curtin N.J. (1998). Potentiation of anti-cancer agent cytotoxicity by the potent poly(ADP-ribose) polymerase inhibitors NU1025 and NU1064. Br. J. Cancer.

[173] Orta M.L., Höglund A., Calderón-Montaño J.M., Domínguez I., Burgos-Morón E., Visnes T., Pastor N., Ström C., López-lázaro M., Helleday T. (2014). The PARP inhibitor olaparib disrupts base excision repair of 5-aza-2′-deoxycytidine lesions. Nucleic Acids Res..

[174] Faraoni I., Consalvo M.I., Aloisio F., Fabiani E., Giansanti M., Di Cristino F., Falconi G., Tentori L., Di Veroli A., Curzi P. (2019). Cytotoxicity and differentiating effect of the poly(ADP-ribose) polymerase inhibitor olaparib in myelodysplastic syndromes. Cancers.

[175] Gaymes T.J., Padua R.A., Pla M., Orr S., Omidvar N., Chomienne C., Mufti G.J., Rassool F.V. (2006). Histone deacetylase inhibitors (HDI) cause DNA damage in leukemia cells: A mechanism for leukemia-specific HDI-dependent apoptosis?. Mol. Cancer Res..

[176] Robert C., Nagaria P.K., Pawar N., Adewuyi A., Gojo I., Meyers D.J., Cole P.A., Rassool F.V. (2016). Histone deacetylase inhibitors decrease NHEJ both by acetylation of repair factors and trapping of PARP1 at DNA double-strand breaks in chromatin. Leuk. Res..

[177] Dellomo A.J., Abbotts R., Eberly C.L., Karbowski M., Baer M.R., Kingsbury T.J., Rassool F.V. (2021). PARP1 PARylates and stabilizes STAT5 in FLT3-ITD acute myeloid leukemia and other STAT5-activated cancers. Transl. Oncol..

[178] Garcia T.B., Snedeker J.C., Baturin D., Gardner L., Fosmire S.P., Zhou C., Jordan C.T., Venkataraman S., Vibhakar R., Porter C.C. (2017). A small-molecule inhibitor of WEE1, AZD1775, synergizes with olaparib by impairing homologous recombination and enhancing DNA damage and apoptosis in acute leukemia. Mol. Cancer Ther..

[179] Faraoni I., Aloisio F., De Gabrieli A., Consalvo M.I., Lavorgna S., Voso M.T., Lo-Coco F., Graziani G. (2018). The poly(ADP-ribose) polymerase inhibitor olaparib induces up-regulation of death receptors in primary acute myeloid leukemia blasts by NF-κB activation. Cancer Lett..

[180] Mufti G., Estey E., Popat R., Mattison R., Menne T., Azar J., Bloor A., Gaymes T., Khwaja A., Juckett M. Results of a phase 1 study of BMN 673, a potent and specific PARP-1/2 inhibitor, in patients with advanced hematological malignancies. Proceedings of the 19th Congress of the European Hematology Association.

[181] Gojo I., Beumer J.H., Pratz K.W., McDevitt M.A., Baer M.R., Blackford A.L., Smith B.D., Gore S.D., Carraway H.E., Showel M.M. (2017). A phase 1 study of the PARP inhibitor veliparib in combination with temozolomide in acute myeloid leukemia. Clin. Cancer Res..

[182] Pratz K.W., Rudek M.A., Gojo I., Litzow M.R., McDevitt M.A., Ji J., Karnitz L.M., Herman J.G., Kinders R.J., Smith B.D. (2017). A phase I study of topotecan, carboplatin and the PARP inhibitor veliparib in acute leukemias, aggressive myeloproliferative neoplasms, and chronic myelomonocytic leukemia. Clin. Cancer Res..

[183] Pratt G., Yap C., Oldreive C., Slade D., Bishop R., Griffiths M., Dyer M.J.S., Fegan C., Oscier D., Pettitt A. (2018). A multi-centre phase I trial of the PARP inhibitor olaparib in patients with relapsed chronic lymphocytic leukaemia, T-prolymphocytic leukaemia or mantle cell lymphoma. Br. J. Haematol..

[184] Fritz C., Portwood S.M., Przespolewski A., Wang E.S. (2021). PARP goes the weasel! Emerging role of PARP inhibitors in acute leukemias. Blood Rev..

[185] Lord C.J., McDonald S., Swift S., Turner N.C., Ashworth A. (2008). A high-throughput RNA interference screen for DNA repair determinants of PARP inhibitor sensitivity. DNA Repair.

[186] Turner N.C., Lord C.J., Iorns E., Brough R., Swift S., Elliott R., Rayter S., Tutt A.N., Ashworth A. (2008). A synthetic lethal siRNA screen identifying genes mediating sensitivity to a PARP inhibitor. EMBO J..

[187] D’Andrea A.D. (2018). Mechanisms of PARP inhibitor sensitivity and resistance. DNA Repair.

[188] Sakai W., Swisher E.M., Karlan B.Y., Agarwal M.K., Higgins J., Friedman C., Villegas E., Jacquemont C., Farrugia D.J., Couch F.J. (2008). Secondary mutations as a mechanism of cisplatin resistance in BRCA2-mutated cancers. Nature.

[189] Swisher E.M., Sakai W., Karlan B.Y., Wurz K., Urban N., Taniguchi T. (2008). Secondary BRCA1 mutations in BRCA1-mutated ovarian carcinomas with platinum resistance. Cancer Res..

[190] Edwards S.L., Brough R., Lord C.J., Natrajan R., Vatcheva R., Levine D.A., Boyd J., Reis-Filho J.S., Ashworth A. (2008). Resistance to therapy caused by intragenic deletion in BRCA2. Nature.

[191] Swisher E.M., Lin K.K., Oza A.M., Scott C.L., Giordano H., Sun J., Konecny G.E., Coleman R.L., Tinker A.V., O’Malley D.M. (2017). Rucaparib in relapsed, platinum-sensitive high-grade ovarian carcinoma (ARIEL2 Part 1): An international, multicentre, open-label, phase 2 trial. Lancet Oncol..

[192] Kondrashova O., Topp M., Nesic K., Lieschke E., Ho G.Y., Harrell M.I., Zapparoli G.V., Hadley A., Holian R., Boehm E. (2018). Methylation of all BRCA1 copies predicts response to the PARP inhibitor rucaparib in ovarian carcinoma. Nat. Commun..

[193] Lin K.K., Harrell M.I., Oza A.M., Oaknin A., Ray-Coquard I., Tinker A.V., Helman E., Radke M.R., Say C., Vo L.T. (2019). BRCA reversion mutations in circulating tumor DNA predict primary and acquired resistance to the PARP inhibitor rucaparib in high-grade ovarian carcinoma. Cancer Discov..

[194] Swisher E.M., Kwan T.T., Oza A.M., Tinker A.V., Ray-Coquard I., Oaknin A., Coleman R.L., Aghajanian C., Konecny G.E., O’Malley D.M. (2021). Molecular and clinical determinants of response and resistance to rucaparib for recurrent ovarian cancer treatment in ARIEL2 (Parts 1 and 2). Nat. Commun..

[195] Hurley R.M., McGehee C.D., Nesic K., Correia C., Weiskittel T.M., Kelly R.L., Venkatachalam A., Hou X., Pathoulas N.M., Meng X.W. (2021). Characterization of a RAD51C-silenced high grade serous ovarian cancer model during development of PARP inhibitor resistance. NAR Cancer.

[196] Pettitt S.J., Krastev D.B., Brandsma I., Dréan A., Song F., Aleksandrov R., Harrell M.I., Menon M., Brough R., Campbell J. (2018). Genome-wide and high-density CRISPR-Cas9 screens identify point mutations in PARP1 causing PARP inhibitor resistance. Nat. Commun..

[197] Bunting S.F., Callén E., Wong N., Chen H.T., Polato F., Gunn A., Bothmer A., Feldhahn N., Fernandez-Capetillo O., Cao L. (2010). 53BP1 inhibits homologous recombination in Brca1-deficient cells by blocking resection of DNA breaks. Cell.

[198] Jaspers J.E., Kersbergen A., Boon U., Sol W., van Deemter L., Zander S.A., Drost R., Wientjens E., Ji J., Aly A. (2013). Loss of 53BP1 causes PARP inhibitor resistance in Brca1-mutated mouse mammary tumors. Cancer Discov..

[199] Wang J., Aroumougame A., Lobrich M., Li Y., Chen D., Chen J., Gong Z. (2014). PTIP associates with Artemis to dictate DNA repair pathway choice. Genes Dev..

[200] Zhou D., Xu P., Zhou X., Diao Z., Ouyang J., Yan G., Chen B. (2021). MiR-181a enhances drug sensitivity of mixed lineage leukemia-rearranged acute myeloid leukemia by increasing poly(ADP-ribose) polymerase1 acetylation. Leuk. Lymphoma.

[201] Fontana D., Ramazzotti D., Aroldi A., Redaelli S., Magistroni V., Pirola A., Niro A., Massimino L., Mastini C., Brambilla V. (2020). Integrated genomic, functional, and prognostic characterization of atypical chronic myeloid leukemia. Hemasphere.

[202] Crisà E., Nicolosi M., Ferri V., Favini C., Gaidano G., Patriarca A. (2020). Atypical chronic myeloid leukemia: Where are we now?. Int. J. Mol. Sci..

[203] Fan J., Li L., Small D., Rassool F. (2010). Cells expressing FLT3/ITD mutations exhibit elevated repair errors generated through alternative NHEJ pathways: Implications for genomic instability and therapy. Blood.

[204] Plo I., Nakatake M., Malivert L., de Villartay J.P., Giraudier S., Villeval J.L., Wiesmuller L., Vainchenker W. (2008). JAK2 stimulates homologous recombination and genetic instability: Potential implication in the heterogeneity of myeloproliferative disorders. Blood.

[205] Al-Ejeh F., Kumar R., Wiegmans A., Lakhani S.R., Brown M.P., Khanna K.K. (2010). Harnessing the complexity of DNA-damage response pathways to improve cancer treatment outcomes. Oncogene.

[206] Chang J., Wang Y., Shao L., Laberge R.M., Demaria M., Campisi J., Janakiraman K., Sharpless N.E., Ding S., Feng W. (2016). Clearance of senescent cells by ABT263 rejuvenates aged hematopoietic stem cells in mice. Nat. Med..

[207] Zhu Y., Tchkonia T., Fuhrmann-Stroissnigg H., Dai H.M., Ling Y.Y., Stout M.B., Pirtskhalava T., Giorgadze N., Johnson K.O., Giles C.B. (2016). Identification of a novel senolytic agent, navitoclax, targeting the Bcl-2 family of anti-apoptotic factors. Aging Cell.

[208] Wyld L., Bellantuono I., Tchkonia T., Morgan J., Turner O., Foss F., George J., Danson S., Kirkland J.L. (2020). Senescence and cancer: A review of clinical implications of senescence and senotherapies. Cancers.

[209] Fleury H., Malaquin N., Tu V., Gilbert S., Martinez A., Olivier M.A., Sauriol A., Communal L., Leclerc-Desaulniers K., Carmona E. (2019). Exploiting interconnected synthetic lethal interactions between PARP inhibition and cancer cell reversible senescence. Nat. Commun..

[210] Saliba A.N., John A.J., Kaufmann S.H. (2021). Resistance to venetoclax and hypomethylating agents in acute myeloid leukemia. Cancer Drug Resist..

[211] Ciccarone F., Valentini E., Bacalini M.G., Zampieri M., Calabrese R., Guastafierro T., Mariano G., Reale A., Franceschi C., Caiafa P. (2014). Poly(ADP-ribosyl)ation is involved in the epigenetic control of TET1 gene transcription. Oncotarget.

[212] Ciccarone F., Valentini E., Zampieri M., Caiafa P. (2015). 5mC-hydroxylase activity is influenced by the PARylation of TET1 enzyme. Oncotarget.

[213] Bamezai S., Demir D., Pulikkottil A.J., Ciccarone F., Fischbein E., Sinha A., Borga C., Te Kronnie G., Meyer L.H., Mohr F. (2021). TET1 promotes growth of T-cell acute lymphoblastic leukemia and can be antagonized via PARP inhibition. Leukemia.

[214] Maifrede S., Le B.V., Nieborowska-Skorska M., Golovine K., Sullivan-Reed K., Dunuwille W.M.B., Nacson J., Hulse M., Keith K., Madzo J. (2021). TET2 and DNMT3A mutations exert divergent effects on DNA repair and sensitivity of leukemia cells to PARP inhibitors. Cancer Res..

[215] Jing C.B., Fu C., Prutsch N., Wang M., He S., Look A.T. (2020). Synthetic lethal targeting of TET2-mutant hematopoietic stem and progenitor cells (HSPCs) with TOP1-targeted drugs and PARP1 inhibitors. Leukemia.

[216] Efficace F., Cottone F., Oswald L.B., Cella D., Patriarca A., Niscola P., Breccia M., Platzbecker U., Palumbo G.A., Caocci G. (2020). The IPSS-R more accurately captures fatigue severity of newly diagnosed patients with myelodysplastic syndromes compared with the IPSS index. Leukemia.

[217] Aoki D., Chiyoda T. (2018). PARP inhibitors and quality of life in ovarian cancer. Lancet Oncol..

[218] Higgins A., Shah M.V. (2020). Genetic and genomic landscape of secondary and therapy-related acute myeloid leukemia. Genes.

[219] Shih A.H., Chung S.S., Dolezal E.K., Zhang S.-J., Abdel-Wahab O.I., Park C.Y., Nimer S.D., Levine R.L., Klimek V.M. (2013). Mutational analysis of therapy-related myelodysplastic syndromes and acute myelogenous leukemia. Haematologica.

[220] Cowell I.G., Austin C.A. (2012). Mechanism of generation of therapy related leukemia in response to anti-topoisomerase II agents. Int. J. Environ. Res. Public Health.

[221] Itzhar N., Dessen P., Toujani S., Auger N., Preudhomme C., Richon C., Lazar V., Saada V., Bennaceur A., Bourhis J.H. (2011). Chromosomal minimal critical regions in therapy-related leukemia appear different from those of de novo leukemia by high-resolution aCGH. PLoS ONE.

[222] Wong T.N., Ramsingh G., Young A.L., Miller C.A., Touma W., Welch J.S., Lamprecht T.L., Shen D., Hundal J., Fulton R.S. (2015). Role of TP53 mutations in the origin and evolution of therapy-related acute myeloid leukaemia. Nature.

[223] Steensma D.P., Bejar R., Jaiswal S., Lindsley R.C., Sekeres M.A., Hasserjian R.P., Ebert B.L. (2015). Clonal hematopoiesis of indeterminate potential and its distinction from myelodysplastic syndromes. Blood.

[224] Bolton K.L., Ptashkin R.N., Gao T., Braunstein L., Devlin S.M., Kelly D., Patel M., Berthon A., Syed A., Yabe M. (2020). Cancer therapy shapes the fitness landscape of clonal hematopoiesis. Nat. Genet..

[225] Lindsley R.C., Saber W., Mar B.G., Redd R., Wang T., Haagenson M.D., Grauman P.V., Hu Z.H., Spellman S.R., Lee S.J. (2017). Prognostic mutations in myelodysplastic syndrome after stem-cell transplantation. N. Engl. J. Med..

[226] Cleven A.H., Nardi V., Ok C.Y., Goswami M., Dal Cin P., Zheng Z., Iafrate A.J., Abdul Hamid M.A., Wang S.A., Hasserjian R.P. (2015). High p53 protein expression in therapy-related myeloid neoplasms is associated with adverse karyotype and poor outcome. Mod. Pathol..

[227] Swisher E.M., Harrell M.I., Norquist B.M., Walsh T., Brady M., Lee M., Hershberg R., Kalli K.R., Lankes H., Konnick E.Q. (2016). Somatic mosaic mutations in PPM1D and TP53 in the blood of women with ovarian carcinoma. JAMA Oncol..

[228] Bolton K.L., Moukarzel L.A., Ptashkin R., Gao T., Patel M., Caltabellotta N., Braunstein L.Z., Aghajanian C., Hyman D.M., Berger M.F. (2020). The impact of poly ADP ribose polymerase (PARP) inhibitors on clonal hematopoiesis. J. Clin. Oncol..

[229] Morton L.M., Dores G.M., Schonfeld S.J., Linet M.S., Sigel B.S., Lam C.J., Tucker M.A., Curtis R.E. (2019). Association of chemotherapy for solid tumors with development of therapy-related myelodysplastic syndrome or acute myeloid leukemia in the modern era. JAMA Oncol..

[230] Shenolikar R., Durden E., Meyer N., Lenhart G., Moore K. (2018). Incidence of secondary myelodysplastic syndrome (MDS) and acute myeloid leukemia (AML) in patients with ovarian or breast cancer in a real-world setting in the United States. Gynecol. Oncol..

[231] Kim G., Ison G., McKee A.E., Zhang H., Tang S., Gwise T., Sridhara R., Lee E., Tzou A., Philip R. (2015). FDA approval summary: Olaparib monotherapy in patients with deleterious germline BRCA-mutated advanced ovarian cancer treated with three or more lines of chemotherapy. Clin. Cancer Res..

[232] Korach J., Turner S., Milenkova T., Alecu I., McMurtry E., Bloomfield R., Pujade-Lauraine E. (2018). Incidence of myelodysplastic syndrome (MDS) and acute myeloid leukemia (AML) in patients (pts) with a germline (g) BRCA mutation (m) and platinum-sensitive relapsed ovarian cancer (PSR OC) receiving maintenance olaparib in SOLO2: Impact of prior lines of platinum therapy. J. Clin. Oncol..

[233] Litton J.K., Rugo H.S., Ettl J., Hurvitz S.A., Gonçalves A., Lee K.-H., Fehrenbacher L., Yerushalmi R., Mina L.A., Martin M. (2018). Talazoparib in patients with advanced breast cancer and a germline BRCA mutation. N. Engl. J. Med..

[234] Banerjee S., Moore K.N., Colombo N., Scambia G., Kim B.G., Oaknin A., Friedlander M., Lisyanskaya A., Floquet A., Leary A. (2021). Maintenance olaparib for patients with newly diagnosed advanced ovarian cancer and a BRCA mutation (SOLO1/GOG 3004): 5-year follow-up of a randomised, double-blind, placebo-controlled, phase 3 trial. Lancet Oncol..

[235] Tew W.P., Lacchetti C., Ellis A., Maxian K., Banerjee S., Bookman M., Jones M.B., Lee J.M., Lheureux S., Liu J.F. (2020). PARP inhibitors in the management of ovarian cancer: ASCO guideline. J. Clin. Oncol..

[236] Wethington S.L., Wahner-Hendrickson A.E., Swisher E.M., Kaufmann S.H., Karlan B.Y., Fader A.N., Dowdy S.C. (2021). PARP inhibitor maintenance for primary ovarian cancer—A missed opportunity for precision medicine. Gynecol. Oncol..

[237] Kayser S., Doehner K., Krauter J., Koehne C.-H., Horst H.A., Held G., von Lilienfeld-Toal M., Wilhelm S., Kuendgen A., Goetze K. (2011). The impact of therapy-related acute myeloid leukemia (AML) on outcome in 2853 adult patients with newly diagnosed AML. Blood.

[238] Martin J., Khalife-Hachem S., Grinda T., Kfoury M., Garciaz S., Pasquier F., Vargaftig J., Uzunov M., Belhabri A., Bertoli S. (2021). Letter to the editor: Therapy-related myeloid neoplasms following treatment with PARP inhibitors: New molecular insights. Ann. Oncol..

[239] Kwan T.T., Oza A.M., Tinker A.V., Ray-Coquard I., Oaknin A., Aghajanian C., Lorusso D., Colombo N., Dean A., Weberpals J. (2021). Preexisting TP53-variant clonal hematopoiesis and risk of secondary myeloid neoplasms in patients with high-grade ovarian cancer treated with rucaparib. JAMA Oncol..

[240] Kartner N., Evernden-Porelle D., Bradley G., Ling V. (1985). Detection of P-glycoprotein in multidrug-resistant cell lines by monoclonal antibodies. Nature.

[241] Schinkel A.H., Mayer U., Wagenaar E., Mol C.A., van Deemter L., Smit J.J., van der Valk M.A., Voordouw A.C., Spits H., van Tellingen O. (1997). Normal viability and altered pharmacokinetics in mice lacking mdr1-type (drug-transporting) P-glycoproteins. Proc. Natl. Acad. Sci. USA.

[242] Erickson-Miller C.L., May R.D., Tomaszewski J., Osborn B., Murphy M.J., Page J.G., Parchment R.E. (1997). Differential toxicity of camptothecin, topotecan and 9-aminocamptothecin to human, canine, and murine myeloid progenitors (CFU-GM) in vitro. Cancer Chemother. Pharmacol..

